# Repair of base damage within break-induced replication intermediates promotes *kataegis* associated with chromosome rearrangements

**DOI:** 10.1093/nar/gkz651

**Published:** 2019-08-08

**Authors:** Rajula Elango, Beth Osia, Victoria Harcy, Ewa Malc, Piotr A Mieczkowski, Steven A Roberts, Anna Malkova

**Affiliations:** 1 Department of Biology, University of Iowa, Iowa City, IA 52245, USA; 2 School of Molecular Biosciences, College of Veterinary Medicine, Washington State University, Pullman, WA 99164, USA; 3 Department of Genetics, Lineberger Comprehensive Cancer Center and Carolina Center for Genome Sciences, University of North Carolina, Chapel Hill, NC 27599, USA

## Abstract

Break induced replication (BIR) is a double strand break repair pathway that can promote genetic instabilities similar to those observed in cancer. Instead of a replication fork, BIR is driven by a migration bubble where asynchronous synthesis between leading and lagging strands leads to accumulation of single-stranded DNA (ssDNA) that promotes mutation. However, the details of the mechanism of mutagenesis, including the identity of the participating proteins, remain unknown. Using yeast as a model, we demonstrate that mutagenic ssDNA is formed at multiple positions along the BIR track and that Pol ζ is responsible for the majority of both spontaneous and damage-induced base substitutions during BIR. We also report that BIR creates a potent substrate for APOBEC3A (A3A) cytidine deaminase that can promote formation of mutation clusters along the entire track of BIR. Finally, we demonstrate that uracil glycosylase initiates the bypass of DNA damage induced by A3A in the context of BIR without formation of base substitutions, but instead this pathway frequently leads to chromosomal rearrangements. Together, the expression of A3A during BIR in yeast recapitulates the main features of APOBEC-induced *kataegis* in human cancers, suggesting that BIR might represent an important source of these hyper-mutagenic events.

## INTRODUCTION

DNA double strand DNA breaks (DSBs) are a dangerous type of DNA damage that if left unrepaired lead to cell death (reviewed in (1)). To avoid such consequences, cells employ multiple DSB repair pathways. One of these repair pathways, break-induced replication (BIR), is employed for repair of DSBs like those formed at collapsed replication forks or by telomere erosion; these types of damage possess only one DNA end capable of invading the homologous template (reviewed in (2,3)). However, repair by BIR comes at a cost to the cell because it also promotes genomic instability (4–8) and in mammals has been implicated in triggering cancer development (9,10). Specifically, overexpression of oncogenes in human cells was shown to promote DNA breakage leading to initiation of BIR resulting in chromosomal rearrangements (9,11). In addition, BIR is responsible for the maintenance of telomeres in ∼10–15% of human cancers through a mechanism called alternative lengthening of telomeres (ALT) (12–14).

Similar to other pathways of homologous recombination, BIR is initiated by 5′ to 3′ DSB end resection (15), resulting in formation of a 3′ single stranded DNA (ssDNA) end, that invades homologous DNA and initiates DNA synthesis (reviewed in (2,3,16)). This repair DNA synthesis, capable of copying hundreds of kilobases, is very different from S-phase replication as, instead of a replication fork, it proceeds via a migrating bubble where asynchronous synthesis between leading and lagging strands results in the accumulation of ssDNA behind the BIR bubble (7,17,18). However, the amount of ssDNA formed during BIR is currently unknown.

This unusual mode of DNA synthesis likely underlies BIR’s 1000-fold elevation in frameshift mutations compared to S-phase replication (6,7). This increased mutagenesis likely results from frequent dissociations of Pol δ from its template during DNA synthesis proceeding in a D-loop context (6). Additionally, BIR occurs in the presence of increased nucleotide pool levels and is associated with inefficient mismatch repair, which also contribute to promoting frameshifts (6).

The likely cause of the >500-fold elevation in base substitutions during BIR (7) is unrepaired DNA damage in the ssDNA accumulated behind the BIR bubble. The frequency of base substitutions during BIR repair can be further increased by exposure of the cells to the DNA alkylating agent methylmethane sulfonate (MMS) (7), highlighting the sensitivity of BIR intermediates to base damage. However, the identity of DNA polymerase(s) responsible for base substitutions during BIR remains unclear. Moreover, the frequency of base substitutions associated with BIR and MMS has been measured only at one position of the BIR track using mutation reporters (7). It therefore remains unclear whether high amounts of mutagenic ssDNA are formed at each part of the BIR track. In addition, mutation clusters (i.e. closely spaced groups of independent mutation events that likely occurred simultaneously), which occur in 50% of BIR events completed in the presence of MMS (19) and are similar to clustered mutations frequently observed in human cancers that are termed *kataegis* (20,21), almost never extended to the chromosome end. This feature suggested that mutagenic ssDNA might never form toward the end of the BIR track, possibly due to conversion of the driving BIR migrating bubble into a normal replication fork. The idea of such conversion that was previously proposed based on the observation of frequent template switching at the beginning BIR, but not at later steps of BIR progression (8), remains untested. Also, the specifics of pathways responsible for the processing of DNA damage induced by MMS could affect the formation and structure of mutation clusters, making it necessary to model BIR-induced *kataegis* using a DNA damage source responsible for *kataegis* associated with cancer.

It is believed that *kataegis* results from the damage initiated in human ssDNA by apolipoprotein B mRNA-editing enzymes catalytic polypeptide 3 enzymes (called APOBEC3′s), which are cytidine deaminases that convert cytidines to deoxyuridines in the context of ssDNA (reviewed in (21–23)). The main function of APOBEC3s in human cells is to restrict viral infections by deamination of mRNA and ssDNA (reviewed in (21–24)). However, besides attacking viral genetic material, APOBEC enzymes can attack human ssDNA causing mutations that might contribute to cancer development (20,25–28). APOBEC3A (A3A) and APOBEC3B (A3B) both are known to predominantly deaminate cytidines in a context of TCA and TCT (collectively TCW) motifs (27,29,30). Cancer cells expressing elevated amounts of A3A or A3B show increased levels of C to T and C to G mutations at TCW motifs (30–33). While A3B is responsible for mutations generated in many cancers, an A3A-induced mutation signature is present particularly in cancers that show the highest mutation load (27). In addition, APOBEC-induced mutations are often associated with gross chromosomal rearrangements (GCRs) (34), although the mechanism of GCR induction in the presence of APOBECs remains unclear.

Since BIR promotes accumulation of ssDNA and is implicated in cancer development, we hypothesized that ssDNA generated during BIR could serve as a substrate for APOBEC to produce mutations and *kataegis*. Here, using yeast *Saccharomyces cerevisiae* as a model, we determine whether mutagenic ssDNA is produced along different positions of the BIR track and we also characterize its potential to promote APOBEC-induced mutagenesis. Using a *ura3-29* reporter we demonstrate similarly high levels of damage-induced mutations at *MAT* locus (near the site of BIR invasion), at 16 kb, and at 90 kb from the site of BIR invasion suggesting that mutagenic ssDNA is formed over the entire track of BIR. Also, we demonstrate that translesion synthesis (TLS) polymerase Pol ζ mediates spontaneous and damage-induced base substitutions resulting from accumulation of ssDNA during BIR. Finally, we report that BIR-associated ssDNA is a potent substrate for the APOBEC3A (A3A) enzyme in yeast, and that this enzyme promotes formation of mutation clusters along the track of BIR. We used *ung1Δ* mutants lacking uracil glycosylase as a tool allowing us to determine the full mutagenic potential of A3A during BIR. Under these conditions, A3A/BIR–induced mutation clusters contained up to 222 mutations in the BIR track. In the presence of uracil glycosylase, the number of mutations in the clusters was reduced due to error-free bypass, which did not rely on Mph1, Ubc13 or on BER enzymes, but proceeded through homologous recombination (HR). However, despite the reduction in mutations, this HR pathway instead stimulated GCRs. Together, the expression of A3A during BIR in yeast recapitulates the main features of APOBEC-induced mutation clusters observed previously in human cancers (i.e. high mutation density and association with GCRs) which suggests that BIR might represent an important initiating factor for *kataegis* formation.

## MATERIALS AND METHODS

### Yeast strains and growth conditions

All yeast strains used in these experiments are isogenic to and are derivatives of AM1003 (Supplementary Table S1) which is disomic for Chromosome III (Chr III) with the following genotype:

*hmlΔ::ADE1/ hmlΔ::ADE3 MATa-LEU2-tel/MATα-inc hmrΔ::HPH FS2Δ::NAT/FS2* *leu2/leu2-3,112 thr4 ura3-52 ade3::GAL::HO ade1 met13*.

AM2889 was derived from AM1003 in several steps. First, *URA3* on Chr. V and *LYS2* on Chr. II were deleted using the *delitto perfetto* approach (35). Second, *LYS2* was inserted between *SED4* and *ATG15*, 36kb from *MAT***a** similar to (6). Third, *hmrΔ::HPH* was replaced by *hmrΔ::KanMX*. In some strain derivatives *HPH*::*Bleo^r^* has been used to replace *hmrΔ::HPH*.

The *ura3-29* reporter was inserted at *MAT* by amplifying the reporter from Ori1 and Ori2 plasmids (36) using the following primers (5′-3′) in which uppercase letters correspond to targeting tails and lowercase letters correspond to sequences in Ori1 and Ori2 plasmids:

TATGTCTAGTATGCTGGATTTAAACTCATCTGTGATTTGTGGATTTAAAAGGTCTTTAATGGGTATTTTATTCATTTTTTagtcagtgagcgaggaagc and TGCTGCATTTTGTCCGCGTGCCATTCTTCAGCGAGCAGAGAAGACAAGACATTTTGTTTTACACCGGAGCCAAACTGTGAGattgtactgagagtgcacc. The *ura3-29* reporter was inserted at position 16kb using the following primers (5′-3′): TCTTTCTGCAATTATTGCACGCCTCCTCGTGAGTAGTGACCGTGCGAACAAAAGAGTCATTACAACGAGGAAATAGAAGAagtcagtgagcgaggaagc and ATATTTGCTGCTATACTACCAAATGGAAAAATATAAGATACACAATATAGATAGTATTAAAAAAACGTGTATACGTTATTattgtactgagagtgcacc (see in (6)). Lastly, the *ura3-29* reporter was inserted at position 90 kb by amplifying the reporter from Ori1 and Ori2 plasmids using the following primers (5′-3′):

ACAACGTTTCCAAAAGTTAGTTAAATACATACGTCTATTTACTAAGCAAGAAATATATCATGACAAGCCCAAATATTATAagtcagtgagcgaggaagc and GCAAGACTACCAGGATCTTTTATCTGATAAGCTCAAATTACCATATTGCTTAATTTCTTACTACTTGTTATAGTGAAAAGattgtactgagagtgcacc

Strains containing *ung1*::*KanMX, rev3::BSD, mph1:: KanMX, ubc13::KanMX, ntg1::KanMX, ntg2::KanMX, rad59::KanMX, mus81::KanMX, KanMX::Bleo^r^, rad30::Bleo^r^*, or *rad1::BSD* disruptions were constructed by transformation with a PCR-derived blasticidin (BSD) marker (TEF/BSD from Invitrogen), *KanMX* marker (37) or phleomycin-resistant *Bleo^r^* marker (38) flanked by terminal sequences matching the first and last 80bp of the open reading frame of each gene (37) and were confirmed by PCR and phenotypic analysis. In addition, *psy3*Δ, *csm2*Δ and *ntg1Δ ntg2Δ apn1Δ apn2Δ* were created by disruption of the *PSY3, CSM2, NTG1* and *NTG2* genes by co-transforming WT or *apn1*Δ *apn2*Δ yeast with the pML107 yeast CRISPR-Cas9 expression vector (39,40) respectively modified to express sgRNAs targeting the sequences (5′-3′): GATGTGATGAAGTTTGACAA, CTTTGTCGGGGAAGGCCAAA, TGAAGAAACTATGGTCAAAC and TGGTTGGACACGGTTACAAA and the following repair templates (5′-3′): TGAAAATTCTTAGGAAAAGAGAAAGGAAGTAGCGAATGGAATGGGTAAGAGAAGGAAAAAAAACATTAAAAACTCAGATACATAAATTAA, AAAATAAAAAAAAAAATGGAGAGAAGAGACTGCTAGCGGCAAAGGCTACATAAGTGCATTTAAAGCATCGGTACACCATGTAACACCAGT, TTGAATGAATAAAAAGTATACATATTAACAACTAGGCCTGCTTTCTTGGGCTATAAAGTATATATAGATACAAATATATGATGAATCATT, and TAATGACATACATAATGGTTTTTGATCTTTTTCCACAATGAATGGTGGTTTATATACTGTGAAAGACGTTGTGCTTATACATTTTTTGTGTGCTCCCAAT.

Finally, *pol3-01* mutant was created by co-transforming *POL3* (wt) strain with CRISPR-Cas9 expression vector bRA89 modified to express sgRNAs targeting the sequences (5′-3′): TCCTTTGATATCGAGTGTGC (41) and the following repair template (5′-3′): CAGCTCCATTGCGTATCATGTCCTTTGCTATCGCGTGTGCTGGTAGGATTGGCGTCTTTCCGGAACCTGAATACGATCCC.

Rich growth media (yeast extract-peptone-dextrose (YEPD) and synthetic complete medium were prepared as described (42). The YEP-lactate (YEP-Lac) and YEP-galactose (YEP-Gal) used for DSB induction were similar to those described in (5). MMS was added to YEP-Gal to the final concentrations of 1.5 mM (similar to (7)). To express APOBEC3A, the yeast were transformed with either A3A-expression or empty vector-control plasmids containing a hygromycin resistance cassette (43) and were grown on YEPD overnight before replica plating to YEPD media supplemented with hygromycin (300 μg/ml) for selection of the plasmid. Yeast cultures were grown at 30°C.

### Analysis of DSB repair

To induce DSBs, galactose was added (final concentration of 2% (w/v)) to a yeast culture that was grown to log phase in YEP-Lac medium. MMS was added to cultures at concentrations of 1.5 mM 30 min after the addition of galactose. Cultures were further incubated at 30°C with agitation for 7 h (similar to (19), which is approximately the time required to complete BIR. MMS was then deactivated by treatment with 10% sodium thiosulfate prior to serial dilution and plating of cells onto YEPD. Cell viability reflects the comparison of the number of colony forming units (CFU) prior to and following 7-hour incubation with galactose in the presence or absence of MMS. Viability was calculated as in (19). Our results demonstrated that cell viability was high (89%) following cell exposure to 1.5 mM MMS. Following DSB induction and repair, the individual DSB repair events (i.e. gene conversion (GC), BIR, chromosome loss, and half crossover (HC)) were identified by phenotypic analysis of colonies that were first grown on YEPD media and then replica plated onto omission media. The percent occurrence of the main DSB outcomes (BIR, GC, chromosome loss and HCs) was calculated by pooling the results from at least three independent experiments per experimental condition that were determined not be statistically significantly different by chi-square test analyses (similar to (5)). To evaluate the presence of rearrangements in chromosome III in association with BIR, we used CHEF gel analysis. BIR events without chromosomal rearrangements contained two copies of chromosome III: a 355 kb chromosome that hybridized to *ADE3*-specific probe and a 345 kb chromosome that hybridized to an *ADE1*-specific probe. BIR outcomes with rearrangements were defined as having two copies of Chr. III where at least one of the copies deviated from its expected size.

### Determining Ura^+^ mutation rates and spectra

The rate of Ura^+^ mutagenesis was determined among all DSB repair outcomes or among Ade^+^ DSB repair outcomes that preserved both copies of chromosome III (6,7). Rates are reported as the median value, and the 95% confidence limits for the median are calculated for the strains with a minimum of six individual experiments (all strains except for *rev3Δrad30Δ*) as described and reported in Supplementary Tables S2 and S3. For *rev3Δrad30Δ* strains with four individual experiments performed, the range of the median was calculated and reported in Supplementary Table S2. Statistical comparisons between median mutation rates were performed using the Mann–Whitney *U* test. The rate of Ura^+^ mutagenesis in the presence of MMS was determined similarly to (7). To determine the rate of Ura^+^ associated with BIR in the presence of A3A expression, the experiments were performed similarly to (7), but 1% Hygromycin (allowing to select for the presence of A3A-containing plasmid) was added in all growth media prior to plating of cells following BIR induction. The analysis of the Ura^+^ mutation spectrum was performed using colony PCR of Ura^+^ outcomes using the following primers (5′-3′):

Primer 1: GTGTGCTTCATTGGATGTTCGTAC and Primer 2:

AAAAGGCCTCTAGGTTCCTTTGTT. The resulting 600 bp-long PCR fragment was Sanger sequenced using the following primer (5′-3′): CTGGAGTTAGTTGAAGCATTAGG (see (7) for details).

### Analysis of clustered mutations by whole-genome sequencing

**Figure 1.**
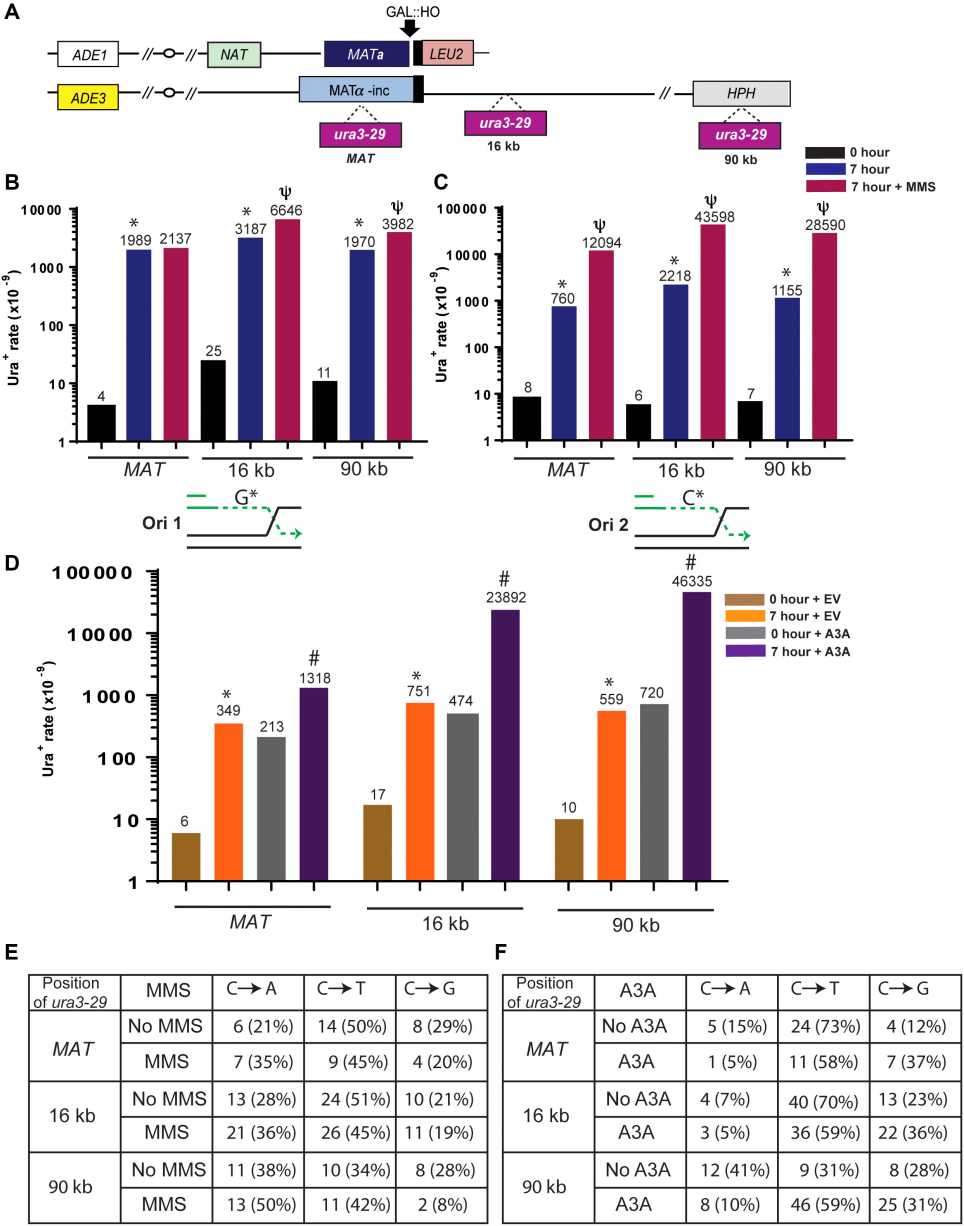
Mutagenic ssDNA accumulates at multiple positions along BIR track. (**A**) BIR is initiated by HO endonuclease at *MAT***a** in a yeast strain disomic for chromosome (Chr) III. Following the DSB, the broken chromosome (top) invades the homologous chromosome (containing *MATα-inc*, bottom) initiating BIR that progresses for ∼100 kb to the end of the chromosome. A base substitution reporter, *ura3-29*, was inserted at different positions of the donor chromosome (0kb (‘MAT’), 16kb, and 90kb away from *MAT*α-inc). (**B**) The rate of Ura^+^ mutations at Ori1-oriented *ura3-29* before (0 hour) and after (7 hours) BIR in the presence (or absence) of 1.5 mM MMS. The median values of the mutation rates calculated for ≥6 experiments are indicated above the bars. Asterisks and psi-symbols indicate statistically significant differences from No-DSB (0 h) and from no-MMS respectively. Absence of symbols indicates no statistical difference between groups. See Supplementary Table S2 for *P*-values, description of statistical analysis, 95% CI of the medians, as well as for BIR efficiencies. Under the graph: schematics illustrating guanine (G*) in the mutant position of Ori1-oriented *ura3-29* included into ssDNA accumulated during BIR leading strand synthesis. (**C**) Ura^+^ mutation rates before and after BIR for Ori2 oriented *ura3-29*, where cytosine (C*) in the mutant position is included into leading strand ssDNA. See (B) for other details. (**D**) Ura^+^ rates for Ori2 *ura3-29* inserted at various Chr. III positions following BIR in the presence of APOBEC3A containing plasmid (A3A) or empty vector (EV). Asterisks and pound-symbols indicate statistically significant differences from No-DSB (0h) and from no-A3A (EV), respectively. See (B) for other details and Supplementary Table S3 for *P*-values, description of statistical analysis, and 95% CI of the medians. (**E**) Mutation spectra of BIR-induced Ura^+^ mutations in Ori2-oriented *ura3-29* in the presence or absence of MMS at various positions of the reporter. (**F**) Mutation spectra of BIR-induced Ura^+^ at Ori2 oriented *ura3-29* in the presence of A3A or empty vector (No A3A).

The preparation of yeast genomic DNA for whole-genome sequencing and library construction were performed similar to (19) and (26). Whole genome sequencing was performed with the HiSeq4000 PE 2 × 150 sequencing platforms. Library preparation was performed using KAPA DNA Hyper kit. Paired end sequencing reads were mapped using CLC Genomic Workbench version 7.5 software (similar to (26,44)) to a reference genome of AM1003 that was created for (19) study, but was tailored here to the AM3617 strain that was isogenic to AM1003, but contained several changes including *ura3-29* reporter inserted into chromosome III (Figure 1A, Supplementary Table S1). Since all isolates displayed >100× sequencing coverage, we required mutations to occur in at least 10 reads. For homozygous mutations in chromosome III and mutations in all other chromosomes, we also required mutations to exist in at least 90% of the total read coverage. Variants on chromosome III with fractional allelic abundances of 27–70% were also included as mutations to enable identification of heterozygous mutations in 2n and 3n regions. All variants occurring in multiple isolates, including sequencing of the AM3617 parental strain, and variants occurring in genomic regions annotated as ‘telomeric repeats’, or ‘LTR’ were excluded from the analysis. The identification of mutation clusters and analyses of strand bias, strand coordination, and co-localization of mutation clusters with breakpoints were performed similar to (19,26). Coverage maps for chromosome III were created using CLC Genomics Workbench version 7.5 software. The assignment of mutations to the recipient or donor copy of chromosome III was performed similar to (7).

## RESULTS

### ssDNA is a source of mutagenesis at multiple positions along the BIR track

To analyze base substitution mutagenesis through the track of BIR, we used our yeast strain, disomic for chromosome III, that contains a galactose-inducible HO endonuclease that can create a DSB at the *MAT***a** locus of the truncated copy of chromosome III (Chr. III) (Figure 1A) (6). The intact (donor) copy of Chr. III serves as a template for repair and contains *MATα-inc* allele that cannot be cut by HO endonuclease (Figure 1A). Distal to the DSB site, all but 46 bp of homology to the donor chromosome has been replaced by *LEU2* and telomere sequences, which makes it possible only for the centromere-proximal DSB end to invade the homologous donor chromosome; this leads to initiation of BIR, the predominant mechanism for the repair of this DSB (5). We have previously demonstrated that in this experimental system initiation of BIR is preceded by extensive 5′-3′ end resection, followed by invasion of the resulting 3′-ssDNA end into the homologous chromosome, often copying the donor chromosome to its end resulting in synthesis tracts of greater than 100 kb (5,6,15). This mechanism of repair leads to conservative inheritance of newly synthesized DNA (7). To test whether mutagenic ssDNA formed during BIR is accumulated at multiple positions along the BIR track, a *ura3-29* base substitution reporter was inserted into three positions in the donor chromosome (Figure 1A). This reporter contains a T to C transition at position 257 in the *URA3* gene, which results in a Phe to Ser amino acid change that produces a non-functional product of *URA3* gene. The *ura3-29* mutation can revert to a Ura^+^ phenotype via C to T, C to G or C to A base substitutions (7,36). We inserted the *ura3-29* reporter into the donor chromosome at three positions relative to the expected location of strand invasion: at *MATα-inc* (‘MAT’), close to the beginning of BIR synthesis, at 16 kb centromere distal to *MAT*, and at 90kb (close to the very end of the chromosome) (Figure 1A). At each of these positions, *ura3-29* was inserted in two orientations, Ori1 and Ori2, which place the mutant guanine or cytosine (respectively) into the ssDNA accumulated during leading strand synthesis of BIR (Figure 1B and C; see schematics under the graphs).

Using this system to assess mutagenesis, we observed highly elevated levels of base substitutions associated with BIR (measured 7 hours following galactose addition) compared to the spontaneous level of base substitutions (no-DSB control; measured at 0 h, prior to addition of galactose), regardless of the position of *ura3-29* within the BIR tract (MAT, 16kb, and 90kb positions) (Figure 1B and C). Moreover, the frequency of base substitutions associated with BIR was nearly indifferent to the orientation of *ura3-29* as the rate of Ura^+^ was increased 95- to-500-fold (as compared to no-DSB level) for different positions in both Ori1 and Ori2 yeast strains (Figure 1B and C; Supplementary Table S2). We therefore conclude that the frequency of base substitutions associated with BIR is increased at multiple positions along the BIR track.

To determine if this elevation in mutagenesis was due to DNA base damage accumulating in the ssDNA formed during BIR, we initially repeated these experiments in the presence of the DNA alkylating agent methyl methansulfonate (MMS), which creates a variety of DNA lesions, including mutagenic N3-methyl cytosine lesions that are specifically formed in ssDNA regions (26,45,46). We observed that the effect of MMS on BIR-associated mutagenesis was orientation dependent. While the effect of MMS on BIR mutagenesis was relatively modest for Ori1, exposure of Ori2 cells undergoing BIR to 1.5 mM MMS led to a further 16- to 25-fold increase of BIR-induced mutagenesis at all *ura3-29* positions. (Figure 1B and C; Supplementary Table S2). In Ori2, the revertible base in *ura3-29* that becomes a part of the ssDNA formed during BIR is a cytosine suggesting that the ssDNA specific N3-methyl cytosine lesion is the source of the increased mutagenesis. In addition, all three types of base substitutions (C to T, C to G, C to A were observed among Ura^+^ revertants in Ori2 strains and their respective fractions were not significantly different between BIR experiments carried out in the absence or presence of MMS or between different reporter positions (Figure 1E). The similar frequencies of mutagenesis observed for both orientations of the mutation reporter following BIR without addition of MMS likely reflect a contribution of different types of DNA damage: those affecting cytosine in ssDNA (similar to MMS) and those affecting guanine in ssDNA (e.g. oxidative damage). Overall, we propose that the elevation in mutagenesis is due to damage accumulated in the ssDNA during BIR.

We next tested whether another ssDNA-specific damaging agent would produce effects similar to MMS by assessing the effect of APOBEC3A (A3A) on BIR-induced mutagenesis. In ssDNA, A3A deaminates cytidine into deoxyuridine (dU), which in turn can be excised by uracil glycosylase to produce a mutagenic abasic (AP) site, ultimately resulting in C to T and C to G substitutions (27). We expressed A3A in our BIR reporter strains with Ori2 oriented *ura3-29* by transforming them with a centromeric plasmid expressing A3A under the control of the TetO7 promoter (39,43). The same plasmid, but without A3A (empty vector), was used as a control. For these experiments, the Ori2 *ura3-29* reporter was chosen for having a cytosine in the revertible base position in the BIR-induced ssDNA (Figure 1C, see schematic), and which is also within a TCT motif, a preferred sequence for deamination by A3A. We observed that expression of A3A led to 4-, 32- and 83- fold increases of BIR-associated mutagenesis at MAT, 16kb, and 90 kb positions, respectively, as compared to no A3A (empty vector) controls (Figure 1D, Supplementary Table S3). In contrast to the spontaneous and MMS-induced spectra (Figure 1E), the A3A-induced Ura^+^ reversions were dominated by C to T and C to G substitutions (Figure 1F). This bias is similar to what was previously reported for APOBEC mutations induced in ssDNA regions formed during DSB resection and telomere erosion (39,47). Thus, we conclude that A3A acts during BIR to induce hyper-mutagenesis by deaminating cytidines in ssDNA accumulated behind the BIR bubble at multiple positions along the BIR track.

### Translesion Polymerase ζ drives mutagenesis promoted by accumulation of ssDNA during BIR

In our earlier study (6), we demonstrated that BIR is associated with highly increased frequency of frameshift mutations, and their formation is driven by Pol δ, the main replicative polymerase during BIR. The DNA polymerase responsible for the increased level of base substitution mutations during BIR was not known. Other studies (46,47) investigating mutagenesis promoted by accumulation of DNA damage in ssDNA have highlighted a role for the translesion synthesis polymerase, Pol ζ.

**Figure 2.**
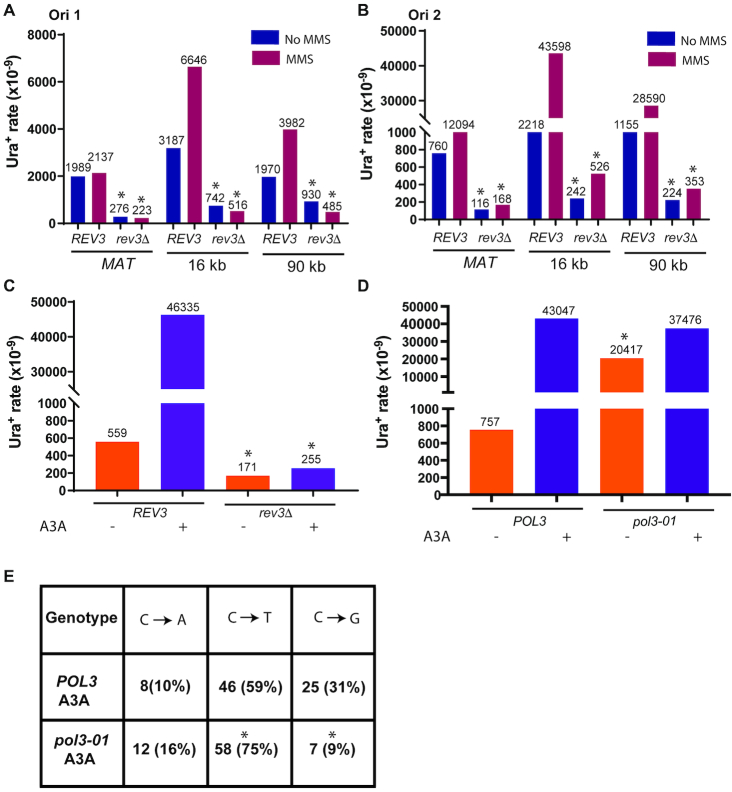
Pol ζ promotes BIR-associated mutagenesis. (**A**) The effect of *rev3Δ* on BIR-associated Ura^+^ rate at Ori1 oriented *ura3-29* inserted at MAT, 16kb, and 90kb positions. The mutation rates were measured in the presence or absence of 1.5 mM MMS. The median values of the mutation rates calculated for ≥6 experiments are indicated above the bars. Asterisks indicate statistically significant differences from *REV3* strains. Absence of symbols indicates no statistical difference between groups. See Supplementary Table S2 for *P*-values, description of statistical analysis, 95% CI or the ranges of the medians, as well as for BIR efficiencies. (**B**) The effect of *rev3Δ* on BIR-associated mutation rates at *ura3-29* in Ori2 orientation at various positions in the presence or absence of MMS. All other details are similar to (A). (**C**) The effect of *rev3*Δ on the rate of BIR-associated mutations in the presence of APOBEC3A (A3A) or empty vector at Ori2 *ura3-29* at 90kb. All other details are similar to (A). The data for *REV3* strains are the same as in Figure 1 and were obtained in parallel with the data for *rev3Δ* strains. (**D**) The effect of *pol3-01* on the rate of BIR-associated mutations at Ori2 *ura3-29* at 90 kb in the presence or absence of A3A. Asterisk indicates statistically significant (*P* = 0.0016) increase of mutations in *pol3-01*-EV as compared to *POL3*-EV. **(E)** Mutation spectra of A3A/BIR-induced Ura^+^ at Ori2 *ura3-29* at 90 kb in *POL3* and *pol3-01* strains. Asterisks indicate statistically significant differences from *POL3* strains for the fraction of C to T (*P* = 0.028) and C to G (*P* = 0.0006) mutations.

To test the role of Pol ζ in base substitution mutagenesis associated with BIR, we deleted the *REV3* gene, which encodes the catalytic subunit of Pol ζ (reviewed in (48)) and assessed the effect of this deletion on the level of Ura^+^ revertants generated during BIR. We observed that the deletion of *REV3* led to a 2- to 9-fold decrease of spontaneous BIR-associated Ura^+^ reversion at every position of the *ura3-29* reporter in both orientations (Figure 2A and B; Supplementary Table S2). An even greater effect of *rev3*Δ was observed with MMS-induced BIR mutagenesis, which was decreased, 8- to 83-fold in the absence of *REV3* (Figure 2A, B; Supplementary Table S2). Finally, deletion of *REV3* led to a 182-fold decrease of A3A-induced BIR mutagenesis (Figure 2C; Supplementary Table S3). Further, we tested whether the residual increase of spontaneous and MMS-induced BIR-associated mutagenesis in the absence of Rev3 could be ascribed to TLS polymerase Pol η. We observed that deletion of *RAD30* (encoding Pol η) in *rev3Δ* strains did not result in further decrease of spontaneous or MMS-induced mutagenesis (Supplementary Figure S1B; Supplementary Table S2). Finally, we observed that the *pol3-01* mutation that eliminates the proofreading activity of Pol δ (49) led to dramatic increase of BIR-induced mutagenesis in the absence of A3A, but did not affect the rate of A3A-induced BIR mutagenesis (Figure 2D). We further tested whether *pol3-01* affects the spectra of A3A/BIR-induced mutagenesis and observed that the fraction of C to G mutations was significantly reduced while the fraction of C to T mutations was significantly increased in *pol3-01* mutants as compared to *POL3* (wild type) strains (Figure 2E). Together, our data demonstrate that Pol ζ mediates most base substitutions occurring during BIR, both in the presence and absence of enhanced DNA damage (i.e. MMS exposure or A3A-expression), while Pol δ likely contributes a number of substitutions due to insertional error.

### An error-free bypass of deoxyuridines during BIR

**Figure 3.**
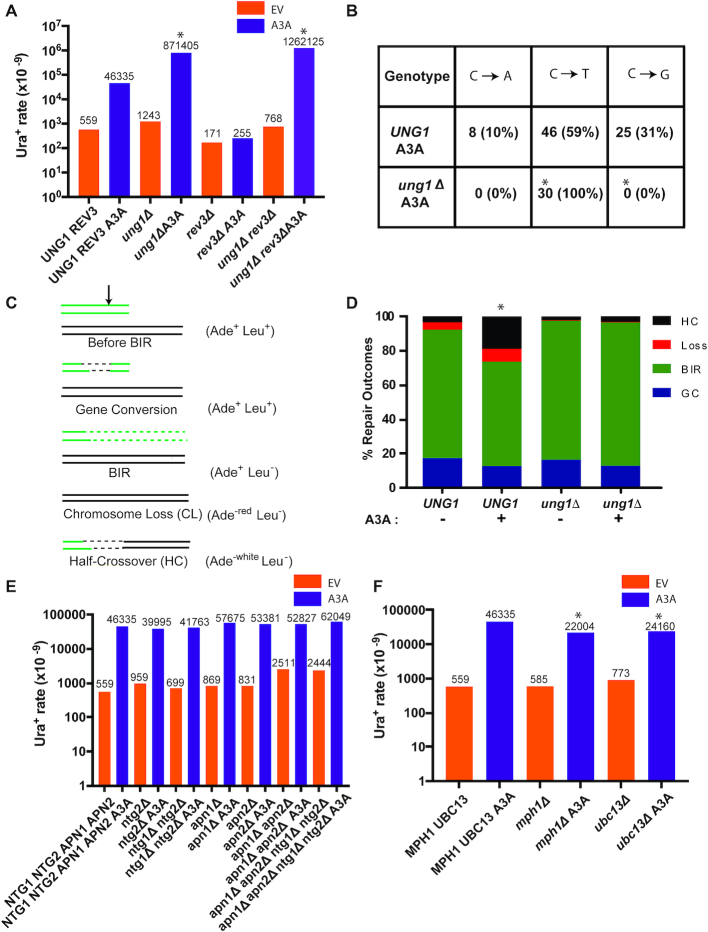
Error-free bypass of abasic sites during BIR. (**A**) The rate of Ura^+^ mutations measured following BIR in the presence of A3A- or empty plasmids in strains bearing Ori2 *ura3-29* at 90kb position. Mutation rates are increased in *ung1Δ* versus *UNG1* in *REV3-*independent way. Asterisks indicate statistically significant differences from *UNG1* strains. See Supplementary Table S3 for *P*-values, description of statistical analysis, 95% CI or the ranges of the medians, as well as for BIR efficiencies. (**B**) Mutation spectra of BIR-associated mutations from (A) in *ung1Δ REV3* A3A and *UNG1 REV3* A3A strains. Asterisks indicate statistically significant differences (*P* < 0.0001) from *UNG1* strains. (**C**) Schematic depicting repair outcomes following DSB induction in yeast disomic strain. Black arrow denotes position of DSB. (**D**) The frequency of half-crossovers (HC) is increased (statistically significant increase is indicated by asterisk) in *UNG1* as compared to *ung1Δ* following DSB induction in the presence of A3A. **Loss**: chromosome loss; **GC:** gene conversion. (**E**) The rates of Ura^+^ following DSB repair in the presence of A3A or empty vector in various base-excision repair mutant backgrounds. The median values of the mutation rates calculated for ≥6 experiments are indicated above the bars. Absence of asterisk indicates no statistical difference between groups. See Supplementary Table S3 for *P*-values, description of statistical analysis, 95% CI or the ranges of the medians, as well as for BIR efficiencies. (**F**) The rates of Ura^+^ following DSB repair in the presence of A3A or empty vector in various mutant backgrounds including *mph1*Δ and *ubc13*Δ. Asterisks indicate statistically significant differences from *MPH1UBC13* strains. All other details are similar to (E). The data for the *wt* strain (*NTG1 NTG2 APN1 APN2 MPH1 UBC13*) are the same as in Figure 1 and Figure 2 and were obtained in parallel with the data for the mutants presented in this figure.

**Figure 4.**
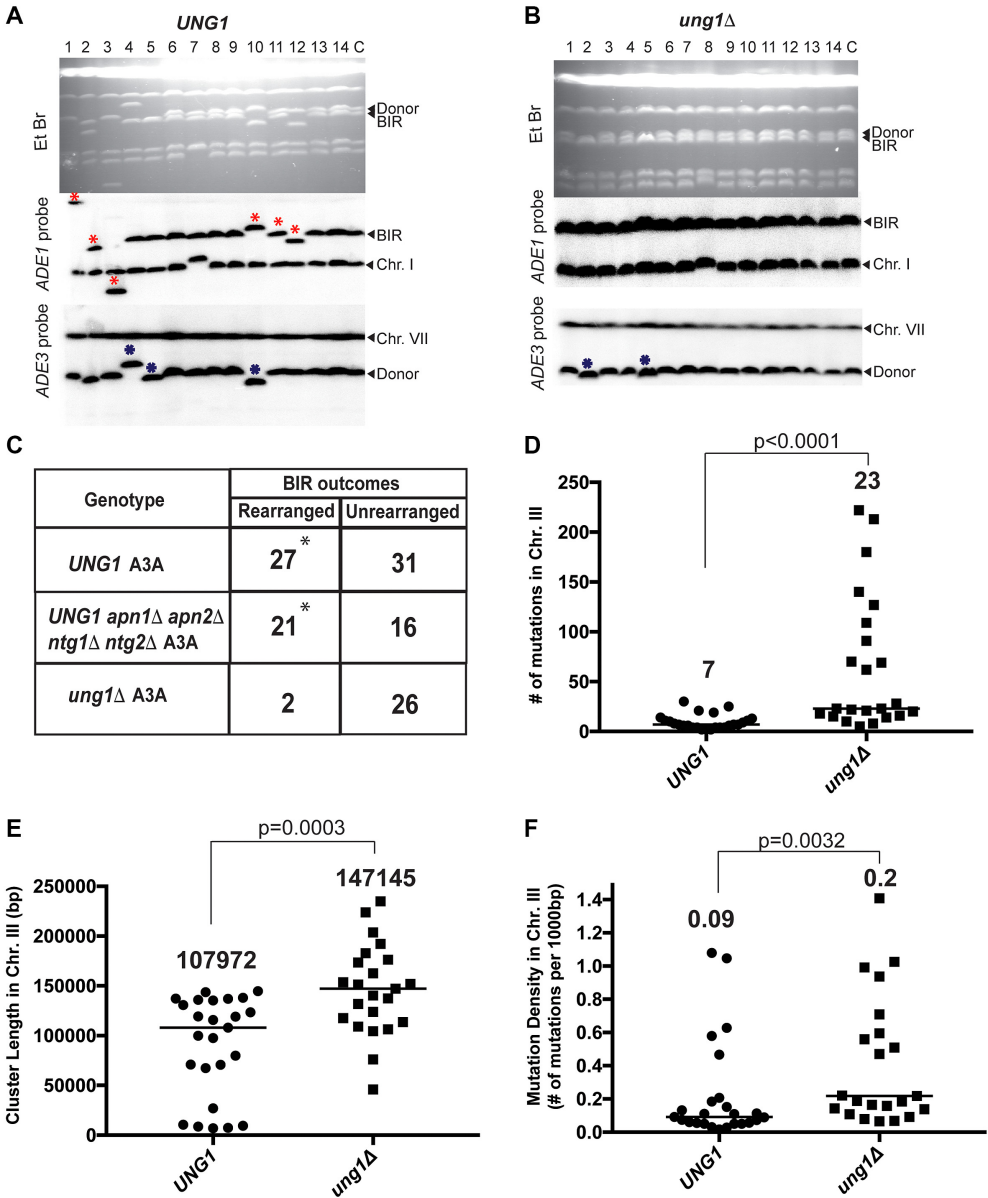
A3A-induced mutation clusters and gross chromosomal rearrangements (GCR) following BIR in *UNG1* and *ung1Δ* strains. (**A**) CHEF gel analysis of Ura^+^ BIR outcomes from *UNG1* and (**B**) *ung1Δ* strains. Top: ethidium bromide-stained CHEF gels; middle: Southern blot hybridization with *ADE1-*specific probe and with *ADE3-*specific probe (bottom). Lane labeled ‘C’: BIR repair control. Lanes labeled 1–14 Ura^+^ BIR outcomes. Red asterisks denote rearranged recipient (*ADE1*-containing) chromosome. Blue asterisks: rearranged donor (*ADE3*-containing) chromosome. (**C**) Rearranged chromosome frequency among Ura^+^ BIR/A3A outcomes in *UNG1*(that is also *APN1 APN2 NTG1 NTG2*), *UNG1 apn1Δ apn2Δ ntg1Δ ntg2Δ* and *ung1Δ* (that is also *APN1 APN2 NTG1 NTG2*). Asterisks indicate statistically significant differences (*P* = 0.0002) from *ung1Δ* strain. (**D**) BIR/A3A-associated mutation clusters in Chr. III detected by whole-genome sequencing (WGS). The number above scatter plots and the line indicate the median numbers of mutations per cluster in *UNG1* and *ung1Δ* strains. *P* values are denoted on top of the line and were determined using the Mann–Whitney *U* test. (**E**) Comparison of BIR/A3A-associated mutation cluster lengths in *UNG1* and *ung1Δ*. The number above scatter plots and the lines indicate the median lengths of BIR/A3A-associated mutation clusters in Chr. III. *P* values for the comparison between *UNG1* and *ung1Δ* are denoted on top of the line and were determined using the Mann–Whitney *U* test. (**F**) Comparison of mutation density (mutations per 1000 bp) in Chr. III mutation clusters in *UNG1* and *ung1Δ*. The median mutation densities are indicated. *P* values for the comparison between *UNG1* and *ung1Δ* are denoted on top of the line and were determined using the Mann-Whitney U test.

The dependence of A3A-induced BIR mutagenesis on Pol ζ indicates that the mutagenic lesion this enzyme causes during BIR is not the dU generated as the result of cytidine deamination because copying of dUs is efficiently accomplished by the replicative polymerases and does not require recruitment of Pol ζ (47,50). We therefore hypothesized that during BIR, the A3A-induced dUs were converted to AP sites by Ung1, resulting in the stalling of Pol δ, and ultimately in Pol ζ-mediated mutagenesis. Moreover, it was possible that Ung1 activity at dU could promote repair of these lesions during BIR. To investigate the involvement of Ung1 activity in the processing of A3A-induced dU formed during BIR, we assessed the frequency of A3A-induced BIR mutagenesis in *ung1Δ* derivatives (i.e. defective for all uracil glycosylase activity) of our strains that contained the Ori2 *ura3-29* reporter at the 90 kb position. We observed that the level of A3A-induced mutagenesis in the absence of *UNG1* was 19-fold higher than in *UNG1* A3A strains (Figure 3A; Supplementary Table S3). Additionally, all Ura^+^ reversions resulting from BIR in *ung1Δ* A3A strains were C to T (Figure 3B), and the high level of Ura^+^ observed in *ung1Δ* A3A strains was independent of Pol ζ (no effect of *rev3Δ*) (Figure 3A; Supplemental Table S3). These results suggest that in the absence of Ung1, dUs persist in BIR-associated ssDNA and lead to very frequent C to T mutations due to incorporation of adenine across from uracils that is likely accomplished by a replicative polymerase mediating lagging strand BIR synthesis. The significant increase in mutagenesis observed in *ung1Δ* A3A (as compared to *UNG1* A3A) (Figure 3A) suggests that AP sites generated by Ung1-mediated excisions of uracil produced by A3A are often (in ∼95% of the cases) repaired or bypassed in an error-free manner. Additionally, CHEF-gel analysis of Ura^+^ BIR outcomes demonstrated that approximately half of Ura^+^ BIR events (27 out of 58) obtained in the *UNG1* A3A strain contained gross chromosomal rearrangements (GCRs; see Materials and Methods for details of GCR analysis). The frequency of GCRs was significantly lower among Ura^+^ outcomes in the *ung1Δ* A3A strain where only 2 of 28 outcomes (7%) were rearranged (Figure 4A–C). An increase in A3A-induced chromosome rearrangements was also supported by the increased frequency of half-crossovers (GCRs resulting from fusions between fragments of BIR donor and recipient chromosomes resulting from resolution or breakage of BIR intermediates (5,51)) in *UNG1* A3A, but not in *ung1Δ* A3A strains (Figure 3C and D). The increase in half-crossovers and other GCRs is indicative of ‘secondary’ recombination events induced by DNA breakage of BIR intermediates in *UNG1* A3A yeast. This ‘secondary’ recombination is potentially a source of AP site error-free bypass in *UNG1* A3A strains.

A recent study (39) demonstrated that AP sites formed in ssDNA during lagging strand synthesis of replication or re-synthesis of eroded telomeres are not repaired by the canonical base excision repair (BER) mechanism. Instead, they are bypassed by error-free template switching involving *MPH1*, which encodes a helicase involved in the error-free template switching pathway (52). In addition, the authors reported that the Rad5-Ubc13-Mms2 ubiquitin conjugating complex involved in the polyubiquitination of PCNA is critical for error-free bypass of replication damage (52–55). In the case of BIR, we observed that neither individual, pairwise, nor quadruple deletion of the genes encoding the only AP lyases or AP endonucleases in yeast (*NTG1* with *NTG2* and *APN1* with *APN2*) significantly affected the level of A3A induced mutagenesis associated with BIR (Figure 3E), indicating that canonical BER does not repair AP sites formed in the ssDNA associated with BIR. However, in contrast to AP sites formed in the lagging strand template during replication or in eroded telomeres, neither deletion of *UBC13* nor deletion of *MPH1* led to an increase of A3A-induced BIR mutagenesis (Figure 3F). In fact, *mph1Δ* and *ubc13Δ* each showed a modest but significant decrease of A3A-induced BIR mutagenesis (Figure 3F; Supplemental Table S3), while the efficiency of BIR in these two mutants was not affected (Supplementary Table S3). In addition, the level of GCRs among Ura^+^ BIR events obtained in the *apn1Δ apn2Δ ntg1Δ ntg2Δ UNG1* strain was indistinguishable from the original *UNG1* strain where all four endonuclease genes remained intact (Figure 4C; Supplementary Figure S1A). This finding supports the existence of another, non-BER pathway(s) that is either fully responsible for DNA breakage at AP sites or may also work in parallel to another pathway, for example in parallel to BER. Deleting *MUS81*, which encodes DNA resolvase that could potentially contribute to DNA breakage (reviewed in (1)) did not increase the level of A3A-induced mutagenesis during BIR in *UNG1* strains (Supplementary Figure S1C, Table S3). In addition, deleting *RAD1* encoding another structure-specific endonuclease (56) in *mus81Δ* cells did not increase the level of A3A-induced mutagenesis either (Supplementary Figure S1C). Similar negative results were obtained after deleting several other recombination genes, including *CSM2* and *PSY3*, that encode DNA binding subunits of the Shu-complex implicated in the bypass of DNA lesions by template switching (57,58), and *RAD59* that encodes a protein required for single-strand annealing (reviewed in (1)) (Supplementary Figure S1C, Table S3), chosen for their possible roles in AP site processing during BIR. Thus, we observed that the involvement of error-free bypass of AP sites was not diminished in our experiments by elimination of various DNA repair pathways; even though we cannot exclude that some of them could contribute redundantly to the error-free bypass.

Overall, we conclude that expression of A3A is synergistic with BIR in promoting base substitutions and GCRs. In addition, the majority of dUs formed by A3A during BIR are processed by uracil glycosylase to form AP sites that are bypassed via an error-free pathway that may involve BER-independent DNA breakage, which is different from the error-free pathways that were previously described for the bypass of AP sites formed during S-phase replication lagging strand synthesis and during filling in of eroded telomeres (39).

### APOBEC3A promotes formation of mutation clusters during BIR

We previously suggested that APOBEC activity on BIR intermediates could be a potent source of *kataegis* in human cancer, which are simultaneously formed localized mutation events (i.e. mutation clusters) frequently associated with complex chromosomal rearrangements. To determine whether A3A stimulates the formation of mutation clusters during BIR, we sequenced the genomes of 50 independent Ura^+^ revertants formed during BIR in the presence of A3A. All these Ura^+^ revertants were obtained in strains containing the *ura3-29* reporter at the 90kb position. Selecting Ura^+^ revertants for whole genome sequencing (WGS) ensured that A3A was expressed in these cells during BIR and also that BIR synthesis reached at least the 90 kb position. Additionally, these 50 sequenced Ura^+^ strains were a subset of 86 Ura^+^ repair events that were previously analyzed by CHEF gel electrophoresis (Figure 4A–C) and selected in such a way that the ratio of events with and without associated GCRs is reflective of what was observed by CHEF. Among 50 Ura^+^ revertants that were selected from this group for WGS, 25 (23 non-rearranged and 2 rearranged) were from *ung1*Δ A3A, while the remaining 25 Ura^+^ (14 non-rearranged and 11 rearranged) were obtained from the *UNG1* A3A strain.

**Figure 5.**
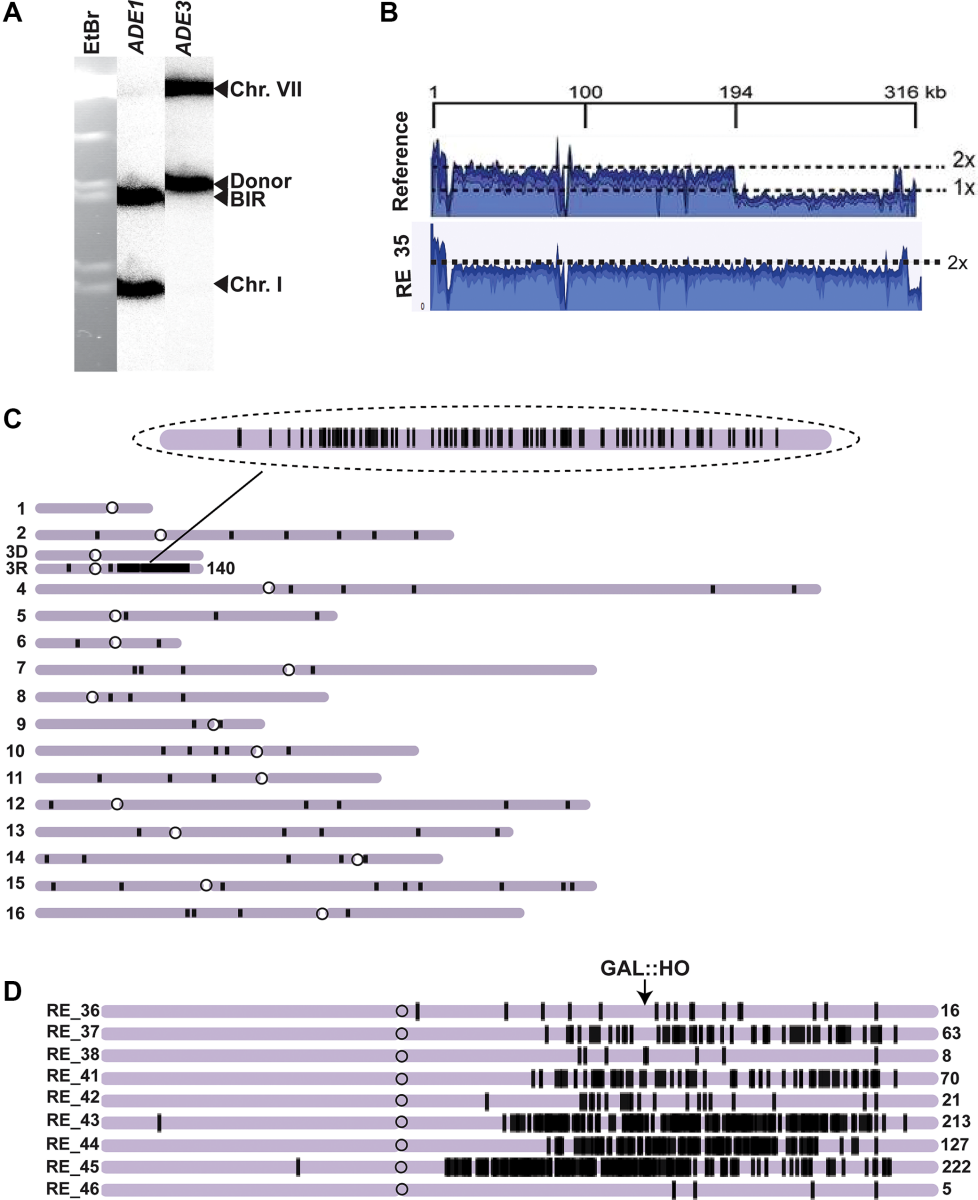
Analysis of mutation clusters induced in *ung1*Δ by BIR/A3A. (**A**) CHEF gel analysis of a representative Ura^+^ BIR outcome (RE_35) isolated in *ung1*Δ. Left: ethidium bromide stained CHEF gel electrophoresis. Middle and right: Southern blot analysis using *ADE1-*and *ADE3-*specific probe, respectively. (**B**) Coverage of Illumina sequencing reads for a BIR event (RE_35) is increased two times for the chromosomal region located centromere-distal to *MAT* (positions >190,180bp) as compared to parental strain. (**C**) A3A-induced mutations (black vertical lines) in RE_35. Enlarged: mutation cluster on the track of BIR (**D**) Clustered mutations in Ura^+^ isolates. Positions of mutations (black lines) are depicted along the chromosome III reference. The total number of mutations in each cluster is indicated by a number (on the right).

To detect and analyze the presence of mutation clusters in *ung1*Δ A3A isolates, we identified groups of mutations where each mutation was no more than 100 kb from the next one and subsequently statistically evaluated whether such groups could be generated by the random distribution of mutations in the genome by comparison to a negative binomial distribution (similar to (26)). Groups of mutations displaying *P*-values <0.01 were categorized as mutation clusters. Analysis of mutations in all 25 *ung1*Δ A3A isolates demonstrated that BIR in every case was associated with the formation of a mutation cluster in the right arm of Chr. III in the area surrounding the HO-cut site (see examples in Figure 5C and D; Supplementary Figure S2–S4, and Table S5). The length of these mutation clusters varied from 46 to 235kb with the median length of the cluster being ∼147kb (Figure 4E). The longest of these mutation clusters was 235 kb long, in RE_35, and it included 140 mutations (Figure 5C, Supplementary Table S5). The median number of mutations per cluster on Chr. III, in the region of BIR comprising <1% of the genome, in *ung1*Δ A3A was 23 (Figure 4D), while the median number of mutations in the remainder of the genome was 28 (Supplementary Table S5). In addition to the presence of one long mutation cluster in the Chr. III region surrounding the HO break site in each isolate, we also identified a total of five mutation clusters in other areas across all 25 *ung1*Δ A3A Ura^+^ strain genomes (Supplementary Table S5). The occurrence of mutation clusters in other chromosomes was not surprising since the expression of A3A in *ung1*Δ strains had previously been reported to promote formation of mutation clusters (39,43). However, the frequency of mutation clusters associated with BIR (affecting <1% of the genome) was higher than in the rest of the genome (*P* < 0.0001). In addition, the median density of mutations in the Chr III (BIR) clusters formed in A3A-expressing *ung1*Δ yeast, ∼1 mutation/5 kb (Figure 4F), was significantly denser (*P* < 0.0001) than in the non-BIR (non-Chr. III) clusters, where it was 1 mutation/11 kb (Supplementary Table S5).

Overall, our data suggest that a combination of A3A expression and BIR leads to the formation of exceptionally dense mutation clusters along the tract of BIR synthesis in *ung1Δ* A3A strains. A3A-promoted BIR mutation clusters occupied large areas of Chr. III, located on both the 5′ and 3′ ends of the DSB (see coverage maps in Figure 5B for coordinates, and Figure 5C and D for distribution of mutations; see also Supplementary Table S5). Mutation clusters on the 5′ end of the break could have resulted from damaged ssDNA that was generated by resection, or BIR synthesis, while mutation clusters on the 3′ end of the break arose from BIR synthesis. The length of mutation clusters on the 5′ end of the HO breakage site (chromosome III position 194 kb (see Figure 5B)) was up to 100 kb (the whole region from *MAT* to the centromere of chromosome III) (see, for example, Figure 5D, RE_45; Supplementary Table S5). This suggests that resection during BIR might proceed for up to 100 kb, which is longer than has ever been reported for BIR (15). Based on the known mechanism of BIR, sequence specificity of A3A, and the absence of uracil glycosylase in the *ung1Δ* strain, we expected that clustered mutations in non-rearranged BIR outcomes would be heterozygous, which would result from deamination of cytosine at TC dinucleotide positions in the Watson strand of chromosome III, resulting in C to T transitions (see e.g. Supplementary Figure S2, RE_26). We observed that out of 1659 mutations in chromosome III mutation clusters detected in *ung1Δ* strains, 1632 occurred in cytosines, and all of them were C to T transitions (Supplementary Table S4), which was consistent with their formation by insertion of adenines across from uracils by a replicative polymerase. Among the 1632 C to T transitions, only 25 were homozygous and could possibly be induced by A3A during S-phase replication, before BIR was initiated (similar to described in (43)), while the rest of them (1607 mutations) were heterozygous and therefore formed during BIR. The role of A3A in the formation of these heterozygous mutations was supported by our observation that 1495/1607 heterozygous mutations occurred at A3A-targeted TC dinucleotides, with 69% of these occurring in the A3A favored TCW trinucleotide motif, where W is either an adenine or thymine (Supplementary Table S4).

Among the two *ung1Δ* isolates that were rearranged, one of them, RE_34 (*ung1*Δ) (Supplementary Figure S3) contained a donor chromosome that was rearranged and became the same size as the repaired BIR chromosome. We propose that in this isolate, two consecutive rounds of BIR synthesis took place. In particular, this repair event was initiated by DSB resection followed by strand invasion between positions *MAT* and *NAT* (located 30kb centromere proximal to *MAT*), followed by initiation of BIR synthesis (primary BIR) (Supplementary Figure S3). This synthesis, which produced clustered mutations in the recipient, continued almost to the end of the chromosome but was interrupted in the region telomere proximal to *ura3-29*. This interruption of ‘primary’ BIR led to the formation of a half-crossover (HC) (similar to (5)) followed by resection and ‘secondary’ invasion by the fragmented donor into the HC at the position centromere-proximal to *NAT*. This led to the initiation of a ‘secondary’ BIR event, allowing *NAT* to be acquired by the donor, and, subsequently, downstream clustered mutations as well. We propose that these clustered mutations in the donor came from two sources: those copied from the HC, which gave rise to homozygous mutations, and those generated during ‘secondary’ BIR synthesis and were therefore heterozygous (refer to schematic in Supplementary Figure S3). For the other rearranged BIR case (RE_31) (Supplementary Figure S4), which was similar in its structure to RE_34 (Supplementary Figure S3) but contained a cluster of heterozygous mutations, we propose that the ‘primary’ BIR synthesis was quickly interrupted producing a HC, while the mutation cluster was formed during ‘secondary’ BIR, when the broken donor copied from the HC.

**Figure 6.**
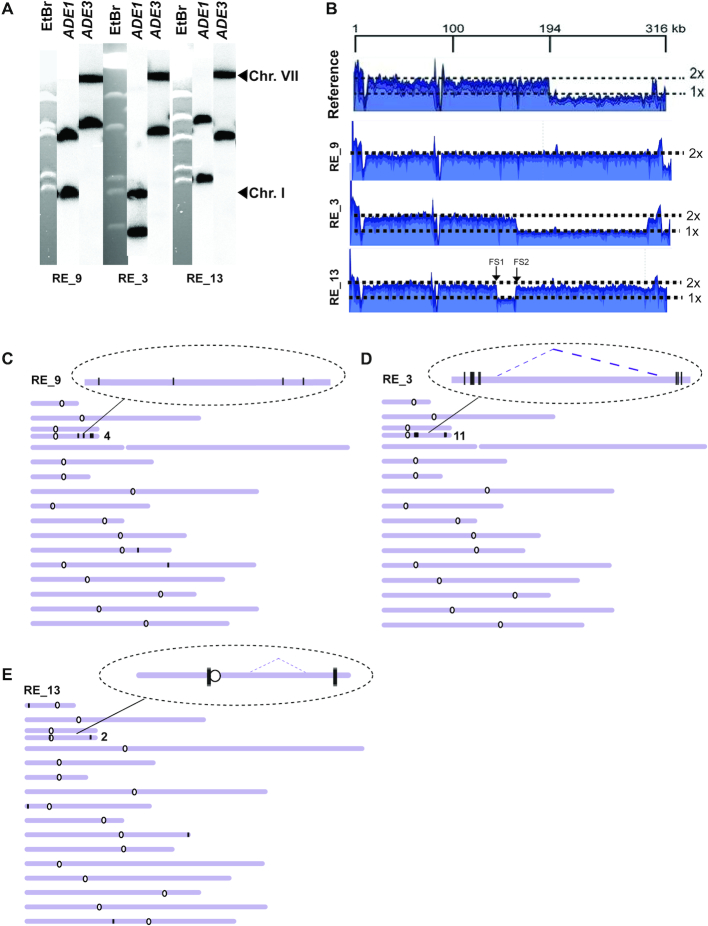
Analysis of mutation clusters induced by BIR in the presence of A3A in *UNG1*. (**A**) CHEF gel analysis of representative Ura^+^ BIR outcomes (Not rearranged: RE_9; Rearranged: RE_3, RE_13). Left: ethidium bromide stained chromosomes separated by CHEF gel electrophoresis. Middle and right: Southern blot analysis using *ADE1-*specific probe and *ADE3-*specific probes, respectively. (**B**) Coverage of Illumina sequencing reads for RE_9, RE_3 and RE_13. 2X indicate fold-increase as compared to the parental strain. (**C–E**) A3A-induced mutations (black vertical lines) in RE_9 (C), RE_3 (D), and RE_13 (E). Enlarged: mutation clusters on the track of BIR. Dotted lines indicate the borders of large deletions. See the legend to Figure 5 for all other details.

We also examined 25 Ura^+^ isolates formed during BIR in the *UNG1* A3A strain by WGS. Our mutation analysis demonstrated that expression of A3A in all of these isolates led to the formation of BIR-associated mutation clusters (located in the right arm of Chr. III) (Supplementary Table S5 and examples in Figure 6). However, the number of mutations in these *UNG1* A3A clusters was significantly lower compared to BIR clusters in *ung1Δ* A3A (*P* < 0.0001) (Figure 4D; Supplementary Tables S4 and S5); also compare mutation clusters in Figure 6C, D and E*(UNG1* A3A) with those in Figure 5C and D (*ung1Δ* A3A). In particular, the median number of mutations in the *UNG1*A3A clusters is 7 with an average of 9 and the median number of mutations in the *ung1Δ* A3A clusters is 23 with the average being 65 (Figure 4D, Supplementary Tables S4 and S5). In addition, the density of mutations in *UNG1* clusters was on average 1 mutation/11 kb, which was significantly lower when compared to that in *ung1*Δ (average of 1mutation/5kb) (*P* < 0.005) (Figure 4F). The lower number of mutations and mutation density in *UNG1* A3A mutation clusters as compared to *ung1Δ* A3A support the idea that the majority of AP sites formed in the *UNG1* A3A background were bypassed during BIR in an error-free way.

Analysis of mutation clusters in the *UNG1* A3A background demonstrates, that 149 out of 184 heterozygous mutations analyzed are in TC motifs targeted by A3A (75% are in the TCW A3A consensus motif) (Supplementary Table S4). The majority of clustered mutations occurred at cytosines on the leading strand, which was similar to what we observed in *ung1Δ* A3A. However, the mutations in *UNG1* were represented by two main classes present in approximately equal amounts: C to T and C to G (Supplementary Table S4 and S5 for WGS data; refer Figure 3B for data using *ura3-29* reporter). This is consistent with their occurrence via a mutagenic bypass of AP sites by translesion polymerase Pol ζ (47,50). In *UNG1* strains expressing A3A, the analysis of coverage maps and mutation clusters in the majority of non-rearranged BIR cases (14/25) suggested that they resulted from a single round of BIR repair (e.g. RE_9, Figure 6A–C and Supplementary Figure S5, RE_6). In accordance, the majority of clustered mutations were heterozygous (Supplementary Table S4, Supplementary Table S5; see schematic in Supplementary Figure S5, RE_6). The only exception among the non-rearranged *UNG1* A3A cases was RE_20 (Supplementary Tables S4 and S5) where BIR was complete (based on coverage map). However, mutations in the BIR cluster in RE_20 were homozygous, which suggested that two rounds of BIR took place, similar to RE_34 in the *ung1*Δ A3A strain (Supplementary Figure S5). The difference between RE_20 and RE_34 is that strand invasion during the ‘second’ BIR event in RE_20 occurred between the regions of *NAT* and *MAT*, which led to no change in the size of the donor.

The elevated frequency of rearrangements in *UNG1*-proficient A3A-expressing strains suggested that A3A-induction of abasic sites in the nascent BIR synthesis tract may also promote more complex rearrangement structures frequently observed in association with *kataegis* events in cancer. We therefore investigated the mechanisms of formation for 11 rearranged BIR events analyzed by WGS in *UNG1* A3A strains by determining their chromosomal structure by CHEF gel analysis in combination with the analysis of coverage maps and the location of clustered mutations obtained by WGS.

Our results suggested that the formation of rearranged BIR outcomes proceeded in general through the following steps: (i) DSB resection and invasion of the recipient into the donor chromosome initiating BIR synthesis (primary BIR), (ii) A3A-promoted deamination of cytosines in ssDNA (resulting from resection or synthesis) leading to accumulation of dU in the recipient, (iii) Ung1-promoted conversion of dU into AP sites, (iv) Stalling of the lagging strand synthesis and/or breakage at the position of the AP site behind the BIR bubble promoting GCR. The exact details of how these steps were completed varied among different repair outcomes, which can explain the variety of phenotypes observed in the outcomes (see the Supplementary Results for details). For example, in a case of three rearranged outcomes, RE_3, RE_11 and RE_21 (Figure 6A, B and D for RE_3), breakage at an AP site behind the BIR bubble likely led to single strand annealing (SSA) between the *TEF* sequences of *NAT* and *Bleo^r^*, which resulted in deletion of a large chromosomal region (refer to schematic in Supplementary Figure S6, Supplementary Table S5, see also Supplementary Results for alternative mechanisms). Similarly, the formation of RE_24 and RE_19 (Supplementary Figure S7, RE_24, Supplementary Table S5) was likely mediated by SSA involving Ty1 elements of *FS1* and *FS2* resulting in the deletion between these two positions (refer to schematic in Supplementary Figure S7 (i)). In the case of RE_1, breakage at an AP site led to invasion into an un-annotated Ty or delta element on the right arm of chromosome II resulting in the formation of translocation (Supplementary Figure S8). We propose that breakage of the newly synthesized leading strand can also explain formation of several other, more complex rearrangements, including RE_13 (Figure 6A, B, E), RE_4 (Supplementary Figure S9), and RE_5 (Supplementary Figure S10), which also involved several rounds of BIR events (see Supplementary Results for the details of these and other events). Overall, based on our WGS analysis, we conclude that mutation clusters are formed in both *UNG1* and *ung1*Δ cells. However, the mutation load is higher in *ung1*Δ whereas *UNG1* cells have a lower mutation frequency but the formation of AP sites in the nascent BIR leading strand often results in the formation of GCRs.

## DISCUSSION

We demonstrate in this work that BIR promotes accumulation of long regions of ssDNA throughout the synthesis tract of this DSB repair pathway. This ssDNA can be targeted by DNA damaging agents, like APOBEC cytidine deaminases, which can lead to high levels of base substitutions and the formation of long and dense mutation clusters. In addition, we determined that DNA polymerase Pol ζ mediates formation of mutations and mutation clusters induced by MMS and APOBEC during BIR. Finally, we propose that an error-free pathway must exist that allows the bypass of DNA damage induced by APOBEC in the context of BIR without formation of mutations. However, this pathway can frequently lead to GCRs, resulting in complex events similar to *kataegis* events observed in cancer.

Interestingly, in the context of BIR proceeding without additional damaging agents, a proofreading defect of Pol δ led to dramatic increase of Ura^+^ reversions (which can possibly result from the increased level of dNTPs during BIR (6)), but it had no significant effect on the frequency of A3A-induced BIR mutagenesis. This suggests that the level of errors made by Pol δ is high when BIR copies an undamaged template, but the majority of these errors are successfully corrected by Pol δ proofreading activity. Consequently, in the presence of functional proofreading by Pol δ, the majority of base substitutions are likely to result from BIR copying of damaged template and are mediated by Pol ζ, which is recruited after stalling of Pol δ during lagging strand BIR synthesis at positions of DNA damage (produced by MMS, APOBEC or spontaneously) in the leading strand. This is similar to how base substitutions arise during other processes (telomere erosion, DSB resection, etc. (47,50)) that also involve accumulation of ssDNA leading to Pol ζ-mediated mutagenesis. The residual mutations that we observed following BIR in *rev3Δ* could result from Pol δ errors that escaped correction by proofreading and mismatch repair (the latter being very inefficient during BIR).

Based on the increase in base substitution frequency at MAT, 16kb, and 90kb positions, we propose that each part of the BIR track (until the end of the chromosome) is present as ssDNA for a considerable period of time. Therefore, we propose that BIR proceeds via migration bubble (promoting accumulation of ssDNA) from its initiation until the end of the chromosome. This would argue against the idea that the migrating bubble driving BIR at the beginning, is later converted into a normal replication fork, which was proposed based on the observation of frequent template switching at the beginning, but not at the end of BIR (8). We propose that this difference likely resulted not from the transitioning to a normal replication fork, but from multiple cycles of invasions, D-loop dissociation or re-invasions occurring only at the beginning of BIR (see in (2,3)). Also, we propose that the formation of interrupted mutation clusters that was observed following BIR in the presence of MMS (19) resulted not from conversion of the migrating bubble into a replication fork, but due to interruption of BIR at positions of MMS-induced template damage. In addition, we observed that the A3A-induced increase of Ura^+^ revertants was lower at the beginning of BIR as compared to the increase that we observed at later steps of BIR (at 16kb and 90kb positions). We propose this difference could be explained by a higher efficiency of error-free bypass of AP sites at the beginning of BIR as compared to later steps of BIR progression.

**Figure 7.**
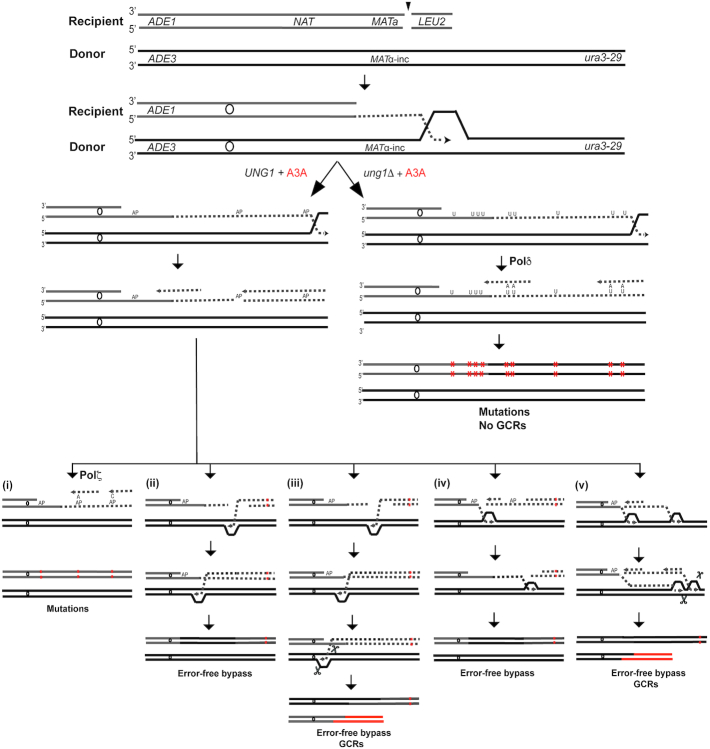
Formation of A3A–induced mutation clusters and GCRs during BIR. DSBs are initiated at *MAT***a**. 5′-3′ resection of DSB ends occurs followed by invasion of 3’ ssDNA ends into the homologous chromosome and initiation of DNA synthesis. A3A deaminates cytidines in ssDNA accumulated during leading strand BIR synthesis, which leads to the formation of uracils (U). Mutations (red asterisks) result from incorporation of adenines (A) across from uracils during lagging strand BIR synthesis in *ung1Δ* (right). In *UNG1* cells, uracil is excised by uracil glycosylase leading to the formation of abasic (AP) site (left). During lagging strand synthesis, Pol δ stalls at AP site, which can lead to the following outcomes: (i) Pol ζ -mediated error-prone bypass of AP site giving rise to C to T or C to G mutations; (ii) Breakage of AP site followed by invasion of 3’ stalled ssDNA end into homologous chromosome followed by initiation of DNA synthesis leading to the error-free bypass of AP site; (iii) DNA synthesis using homologous chromosome is initiated similar to (ii), but interrupts due to collision at some replication obstacle (e.g. centromere) resulting in half-crossover and GCRs; (iv) Breakage at AP site leads to the invasion of another (centromere-proximal) 3’-ssDNA end into the homolog followed by DNA synthesis proceeding in the direction of telomere and resulting in error-free bypass of AP sites or (v) Collision of error-free bypass synthesis with BIR bubble leading to interruption of DNA synthesis and formation of GCRs.

The expression of A3A cytidine deaminase dramatically increased base substitutions and promoted the formation of mutation clusters along the track of BIR. The presence or absence of functional uracil glycosylase define two pathways of A3A-induced DNA damage processing during BIR. In *ung1Δ* cells lacking uracil glycosylase, deamination of cytidines by A3A in ssDNA formed during BIR leads to accumulation of uracils in DNA that are not processed further (Figure 7, *ung1Δ* (right)). During lagging strand BIR synthesis, a replicative polymerase (likely Pol δ), incorporates adenines across from these uracils, which leads to the formation of C to T transitions through the entire track of BIR. This is similar to the mechanism of C to T mutations induced by APOBECs described in other yeast systems in the absence of uracil glycosylase (39,43,59). The length and density of the mutation clusters that we detected in *ung1Δ* A3A cells reflect events over the total length of ssDNA formed during individual BIR events and that resulted from BIR synthesis and resection, which is long and in some exceptional cases proceeded for up to 100 kb. We observed that every Ura^+^*ung1Δ* A3A BIR outcome contained a mutation cluster; these clusters varied in their lengths and mutation density, which we presume reflects variation in the extent of DSB resection, persistence of ssDNA and/or in the level of A3A expression.

In *UNG1* A3A cells, nearly all uracils formed by A3A during BIR are converted into AP sites by uracil glycosylase. This is consistent with the strong dependence of BIR/A3A induced mutagenesis in the *UNG1* strains on Pol ζ. It is also supported by a significant fraction of C to G (along with C to T) base substitutions among mutations. This is similar to what was reported for mutagenesis induced by APOBEC3B at eroded telomeres in baker's yeast (39). It differs from what was reported in association with lagging strand DNA replication (39), where expression of APOBEC3B led to a much higher frequency of C to T transitions as compared to C to G transversions, and there was no strong dependence of mutagenesis on Pol ζ (39). The difference observed between APOBEC-induced mutagenesis at eroded telomeres versus lagging strand synthesis was explained by the greater persistence of ssDNA at eroded telomeres, which allowed conversion of nearly all uracils into AP sites, and therefore required Pol ζ for mutagenesis (39). In contrast, ssDNA formed during lagging strand synthesis was short-lived and allowed some uracils to serve directly as templates for Pol δ, resulting in C to T mutations (39). We propose that ssDNA formed during BIR is highly persistent, which allows the vast majority of uracils to be converted into AP sites. These AP sites likely promote stalling of Pol δ during lagging strand BIR synthesis. Mutations result from TLS bypass of AP sites where Rev1 or Pol δ insert a nucleotide across from AP site, while Pol ζ extends the inserted nucleotide (Figure 7(i)). The increase of C to T and the decrease of C to G mutations in the absence of Pol δ proofreading indicates that Pol δ predominantly inserts adenine across from AP site. This is the first evidence to our knowledge of Pol δ proficiency to insert a nucleotide across from an AP site. Further, our data suggest that proofreading activity of Pol δ can remove this insertion, which allows Rev1 to insert cytosine. Thus, the ratio of C to T to C to G depends on the ratio of Pol δ-versus Rev1-mediated insertions.

We believe that the 19-fold decrease in mutation frequency that we observed *in UNG1* A3A as compared to *ung1Δ* A3A indicates that mutagenic events in *UNG1* A3A strains occur at only ∼5% of the AP sites, while ∼95% of AP sites are bypassed in an error-free way (Figure 7). This error-free bypass differs from the Mph1/Ubc13-dependent template switching that mediates error-free bypass of AP sites during S-phase replication and during filling-in of eroded telomeres (39). Deletion of *MPH1* or *UBC13* surprisingly caused a slight decrease in mutagenesis in our experiments. This might result from the increase of BIR processivity reported previously for *mph1Δ* mutants (60). Also, this error-free bypass observed in our experiments did not involve BER enzymes (Apn1, Apn2, Ntg1 or Ntg2) that are known to contribute to repair of AP sites introduced during tRNA gene transcription (59). We propose that during BIR, the bypass of AP sites involves homologous recombination initiated by strand invasion of the 3′ stalled DNA end into the BIR donor (Figure 7(ii) and (iii)). We propose that initiation of this recombination may be preceded by breakage of DNA at the AP-site that can occur spontaneously or by enzymatic cleavage. Since we did not observe any increase of mutagenesis or decrease of GCR level in the absence of Apn1, Apn2, Ntg1, and Ntg2 enzymes, we favor the idea of spontaneous breakage that was reported for AP sites under the condition of increased temperature (61,62). It is also possible that several different mechanisms of AP site error-free bypass work in parallel with each other, which makes their identification difficult. In any case, when the stalled 3′end invades the homologous chromosome, the repair synthesis is expected to proceed towards the centromere in our system (Figure 7(ii) and (iii)). Such synthesis might not be as mutagenic as the primary leading strand BIR synthesis because the displaced newly synthesized DNA produced during bypass synthesis is expected to anneal to the leading strand immediately and therefore will not accumulate as ssDNA (Supplementary Figure S5). Thus, we propose that recombination bypass of AP sites is a key mechanism of mutation avoidance during BIR. Interruption of this recombination bypass synthesis might promote resolution of bubble-migration intermediates leading to the breakage of the donor chromosome followed by formation of GCRs (Figure 7(iii)) similarly to what we previously described for the formation of half-crossovers and half-crossover initiated cascades (51). The reasons for interruption might include colliding with difficult-to-replicate sequences, for example a centromere (if replication was initiated towards the centromere). Alternatively, bypass DNA synthesis might be initiated in the opposite direction (in the case of DSB formation and strand invasion of the 3′ end located on the centromere proximal DSB side (Figure 7(iv) and (v)). In this case, DNA synthesis will proceed in the direction of the telomere and might be interrupted by collision with the primary BIR bubble moving in the same direction (Figure 7(v)). We propose that interruptions of bypass DNA synthesis leads to the resolution of secondary bubble intermediates, chromosome breakages followed by GCRs that we frequently observed among *UNG1* outcomes and almost never among *ung1Δ* outcomes. Overall, we propose that during lagging strand BIR synthesis, Pol δ stalls at the position of AP site, and the repair is channeled into error-free bypass by ‘secondary’ recombination, which enables avoidance of point mutations, but leads to GCRs. The predominance of error-free over TLS bypass of AP sites during BIR is supported by our observation that *pol3-01* (proofreading mutation) did not affect the frequency of mutations in *UNG1* strains even though it affected their spectrum. Using yeast as a model, Poltoratsky and Pavlov (63) previously demonstrated that AID (an APOBEC-like enzyme)-induced damage stimulates recombination in *UNG1*-dependent manner. However, it remained unclear whether in addition to increased allelic recombination this damage also stimulated GCRs. It will be important to determine whether induction of GCRs is specific to APOBEC damage induced in a context of BIR or can occur in other contexts as well. It is possible that the long track of DNA synthesis or the possibility of collision between several migration bubbles can potentially make BIR unique in this respect.

Extrapolating from our results in yeast, we propose that BIR can be a prominent source of APOBEC-induced mutation clusters in cancer cells. Importantly, our data suggest that a high frequency of GCRs that have been previously associated with A3A-induced mutation clusters (reviewed in (21)) can be explained by the damage conversion and bypass of AP sites during BIR that we described here. It is possible that frequent association with GCRs will become a distinguishing feature of BIR-associated APOBEC-induced mutation clusters. The induction of BIR in cancer cells has been recently documented by (9) who observed that overexpression of oncogenes in human cancer cells leads to collapse of replication forks, which promotes BIR that leads to chromosome rearrangements (9–11,64). In addition, 10–15% of all cancers use alternative lengthening of telomeres (ALT) proceeding via a BIR-like mechanism to maintain their telomeres (reviewed in (12)). These BIR events could lead to the formation of APOBEC-induced mutation clusters associated with GCRs. Our results indicate that the actual amount of APOBEC-induced damage taking place in the BIR synthesis tract was underestimated when measuring the length and density of mutation clusters in *UNG1* cells, and the loss of uracil glycosylase activity that can occur in cancer cells (65,66) would lead to the formation of exceptionally long mutation clusters with high mutation density. We expect that future studies will confirm the involvement of BIR in the formation of mutation clusters in human cancers.

## DATA AVAILABILITY

Raw sequencing reads can be accessed from the NCBI Sequence Read Archive database under bioproject accession number PRJNA517571.

## Supplementary Material

gkz651_Supplemental_Files

## Notes

Present address: Rajula Elango, Department of Medicine, Division of Hematology-Oncology and Cancer Research Institute, Beth Israel Deaconess Medical Center and Harvard Medical School, Boston, MA, USA.
