# Supplementary material for: Repair of base damage within break-induced replication intermediates promotes kataegis associated with chromosome rearrangements
Source: Nucleic Acids Res. 2019 Aug 8;47(18):9666–84. doi: 10.1093/nar/gkz651 (PMC6765108; doi:10.1093/nar/gkz651)
Supplement: gkz651_Supplemental_Files [file gkz651_supplemental_files.zip › Supplementary Results and Figures.pdf]

SUPPLEMENTARY RESULTS

Characterization of mutation clusters in BIR isolates.

To determine molecular mechanisms for the formation of mutation clusters, we used various approaches including phenotypic analysis, contour-clamped homogeneous electric field (CHEF) electrophoresis and whole genome sequencing. Below, we present the details of analysis for representative Ura^{+} isolates obtained from *ung1Δ* and *UNG1* following BIR in the presence of A3A expression.

Characterization of *ung1Δ* isolates.

Characterization of RE_26

RE_26 is an Ade^{+} Leu^{-} Ura^{+} non-rearranged BIR outcome containing a 153,508 bp mutation cluster overlapping the track of BIR. Coverage of Illumina sequencing reads for RE_26 is increased two times (2X) for the chromosomal region located centromere-distal to *MAT* (positions >190,180 bp) as compared to the reference (Supplementary Figure S2A). Using CHEF, we observed that RE_26 contained a BIR product (hybridizing to *ADE1*-specific probe) that was 345kb in size and a donor chromosome (hybridizing to *ADE3*-specific probe) that was 355kb in size (Supplementary Figure S2B). We propose that the formation of RE_26 included the following steps. First, following DSB induction and 5' to 3' DSB end resection, the 3' single strand end invaded the homologous donor chromosome centromere-distal to *NAT*. Following invasion, BIR synthesis led to the accumulation of a long region of ssDNA behind the bubble, where A3A converted cytidines into uracils (U). The insertion of adenines across from uracils during lagging strand synthesis led to the formation of the mutation cluster. The mutations in this cluster located centromere-proximal from *MAT* could result from ssDNA formed during DSB end resection or from BIR synthesis (Supplementary Figure S2C).

Characterization of RE_34

RE_34 is an Ade^{+} Leu^{-} Ura^{+} BIR isolate showing 2X coverage of Illumina sequencing reads for the chromosomal region located centromere-distal to *MAT* (Supplementary Figure S3A). This isolate contains a mutation cluster in chromosome III accompanied by a GCR. It contains a BIR product (chromosome hybridizing to *ADE1*-specific probe) and a donor chromosome (hybridizing to *ADE3*-specific probe) that are both 355kb in size (Supplementary Figure S3B). We propose that the formation of RE_34 was initiated by invasion of the broken recipient into the donor chromosome centromere-distal to *NAT* followed by BIR synthesis that proceeded almost to the end of the chromosome where it was interrupted, which led to a half-crossover (HC) event. Following the formation of the half-crossover, the mutations that arose during the first round of DNA synthesis ended up in the recipient chromosome. Next, the broken donor chromosome underwent extensive 5' to 3' resection, and invaded the recipient chromosome (HC) centromere-proximal to *NAT*. This led to the initiation of a "secondary" BIR event, allowing *NAT* to be acquired by the donor, and, subsequently acquiring downstream clustered mutations as well. We propose that clustered mutations in the donor of RE_34 came from two

sources: those copied from the HC, which gave rise to homozygous mutations, and those generated during "secondary" BIR synthesis that were therefore heterozygous (refer to schematic in Supplementary Figure S3C).

Characterization of RE_31

RE_31 is an Ade$^+$ Leu$^-$ Ura$^+$ isolate with 2X coverage of Illumina sequencing reads for the chromosomal region located centromere-distal to *MAT* (Supplementary Figure S4A). This isolate contains a mutation cluster in chromosome III accompanied by a GCR. It contains a BIR product (chromosome hybridizing to *ADE1*-specific probe) and a donor chromosome product (hybridizing to *ADE3*-specific probe) that are both 355kb long (Supplementary Figure S4B). RE_31 is similar in its structure to RE_34 (Supplementary Figure S3) but contains a cluster of heterozygous mutations. We propose that the formation of RE_31 was initiated by invasion of the recipient into the donor chromosome centromere-distal to *NAT.* This synthesis was quickly interrupted producing a HC. The broken donor underwent resection, which produced ssDNA in the donor chromosome. This ssDNA was susceptible to deamination by A3A producing uracils (U). The 3' ssDNA broken end of the resected donor invaded into the recipient chromosome centromere-proximal to *NAT* and initiated a "secondary" BIR event. This secondary BIR used the recipient (HC) as a template and continued DNA synthesis till the end of the chromosome, which led to the formation of the donor that was identical in size to the HC and contained a cluster of predominantly heterozygous mutations (Supplementary Figure S4B and C).

Characterization of *UNG1* isolates.

Characterization of RE_6

RE_6 is an Ade$^+$ Leu$^-$ Ura$^+$ non-rearranged BIR outcome with 2X coverage of Illumina sequencing reads for the chromosomal region located centromere-distal to *MAT* (Supplementary Figure S5A). It contains a BIR product (chromosome hybridizing to *ADE1*-specific probe) that is 345kb long and a donor chromosome (hybridizing to *ADE3*-specific probe) that is 355kb long (Supplementary Figure S5B). We propose that following DSB resection, the DSB end invaded the homologous donor centromere-distal to *NAT* and initiated DNA synthesis that proceeded all the way till the end of the chromosome. During BIR synthesis, ssDNA was accumulated in the leading strand, and A3A promoted deamination of cytosines in this ssDNA leading to accumulation of U in the recipient. Next, Ung1 promoted conversion of U into AP-sites. Stalling of the lagging strand synthesis and/or breakage at the position of the AP-site behind the BIR bubble promoted error-free bypass of AP-sites *via* a secondary HR event initiated by invasion into the donor chromosome to be used as a template. Alternatively, TLS bypass of AP-sites promoted formation of a mutation cluster (Supplementary Figure S5C).

Characterization of RE_3

RE_3 is an Ade$^+$ Leu$^-$ Ura$^+$ BIR outcome with 2X coverage of Illumina sequencing reads for the chromosomal region located centromere-distal to *MAT* and contains a mutation cluster accompanied by a GCR (Supplementary Figure S6A). It contains a rearranged BIR product

(chromosome hybridizing to *ADE1*-specific probe) that is 217kb long and an intact donor chromosome (hybridizing to *ADE3*-specific probe) that is 355kb long (Supplementary Figure S6B). We propose that during the formation of this isolate, BIR leading strand synthesis proceeded until the very end of the chromosome, as reflected by the cluster of heterozygous mutations formed at the chromosome end (Supplementary Figure S6C). Next, DNA breakage occurred at the position of an AP-site introduced into the ssDNA produced by the leading strand behind the BIR bubble, which was repaired by single strand annealing (SSA) between the *TEF* promoter sequences of *NAT* (located at position 167kb of Chr. III) and of *BLEO*^{r} (a part of *ura3-29* insertion, located at the 290kb position). This led to deletion of a large chromosomal region between these two positions (Supplementary Figure S6C). Alternatively, it is possible that following the break, the broken fragment of the recipient chromosome initiated secondary BIR by strand invasion of the *TEF* sequence of *NAT* into the *TEF* sequence of *BLEO*^{r}.

Characterization of RE_24

RE_24 is an Ade^{+} Leu^{-} Ura^{+} BIR outcome with 2X coverage of Illumina sequencing reads for the chromosomal region located centromere-distal to *MAT* and contains a mutation cluster accompanied by a GCR. In particular, the chromosome III region between *FS1* and *FS2* showed only 1X coverage of Illumina sequencing reads indicative of deletion of this region (Supplementary Figure S7A). The recipient chromosome (chromosome hybridizing to *ADE1*-specific probe) was 225kb long (consistent with a ~20kb deletion between the *FS1* and *FS2* regions). The donor chromosome (hybridizing to *ADE3*-specific probe) was 355kb long (Supplementary Figure S7B). We envision that normal BIR leading strand synthesis initiated by strand invasion of the broken recipient chromosome end centromere-proximal to *FS2* (Supplementary Figure S7C) led to the formation of A3A-induced U's being converted into AP-sites by uracil glycosylase. Next, breakage at the position of an AP-site in the newly synthesized leading strand led to SSA involving Ty1 elements *FS1* and *FS2,* and resulted in the deletion between these two positions (refer to schematic in Supplementary Figure S7C (i)). Another possibility is that deletion resulted from a template switching event that occurred during lagging strand synthesis between positions of Ty1 elements *FS2* and *FS1* (Supplementary Figure S7C (ii)).

Characterization of RE_1

RE_1 is an Ade^{+} Leu^{-} Ura^{+} outcome with a mutation cluster in chromosome III and 1X coverage of Illumina sequencing reads for the chromosomal region located centromere-distal to *FS1.* In addition, this isolate showed a 2X coverage of the right arm of chromosome II (Supplementary Figure S8A), indicative of a translocation involving chromosomes II and III. The recipient chromosome III (hybridizing to *ADE1*-specific probe) is 217kb long and the donor chromosome III (hybridizing to *ADE3*-specific probe) remained intact and is 355kb long (Supplementary Figure S8B). We propose that the formation of RE_1 resulted from DNA breakage at an AP-site formed in the leading BIR strand, which led to the loss of most of the newly synthesized BIR strand and the re-invasion of Ty1 of *FS1* of the recipient into a un-annotated Ty or delta element, located close to the centromere on the right arm of chromosome II (Supplementary Figure S8C). The absence of mutation clusters in chromosome II of RE_1 indicates that BIR synthesis using

chromosome II as a template was quickly interrupted, which led to the formation of a translocation resulting from half-crossover (Supplementary Figure S8C).

Characterization of RE_4

RE_4 is an Ade^+ Leu^- Ura^+ BIR outcome with 2X coverage of Illumina sequencing reads for the chromosomal region located centromere-distal to *MAT*, and 3X coverage of the region between *FS1* and *FS2* (Supplementary Figure S9A). The recipient chromosome (hybridizing to *ADE1*-specific probe) is 345kb in size, while the donor chromosome (hybridizing to *ADE3*-specific probe) is rearranged and is approximately 375kb long (Supplementary Figure S9B). The rearrangement in RE_4 is the most complex and we propose that this event likely resulted from a "secondary" BIR event initiated after the collision of the primary BIR bubble and a repair bubble formed following breakage at an AP-site, which led to breakage of the donor chromosome. The donor chromosome then proceeded to resect, followed by invasion of *FS2* of the donor into *FS1* of the recipient and copying of the recipient chromosome. This led to an increase in the size of the donor chromosome resulting from the additional synthesis of the region between *FS1* and *FS2* (Supplementary Figure S9C).

Characterization of RE_5

RE_5 is an Ade^+ Leu^- Ura^+ BIR outcome with 2X coverage of Illumina sequencing reads for the chromosomal region located centromere-distal to *MAT* (Supplementary Figure S10A). This isolate contains heterozygous mutations accompanied by GCRs. It shows a BIR product (chromosome hybridizing to *ADE1*-specific probe) and a donor chromosome (hybridizing to *ADE3*-specific probe) that are both 355kb in size (Supplementary Figure S10B). We propose that formation of RE_5 was initiated by invasion of the recipient into the donor chromosome centromere-distal to *NAT*. This synthesis was interrupted towards the end of the chromosome presumably by collision of a secondary migrating bubble arising from breakage at an AP-site with the primary bubble, and resolution into a HC event. The broken donor then resected extensively, invaded the intact recipient, and copied until the end of the chromosome, thereby producing heterozygous mutations (Supplementary Figure S10C).

Supplementary Table S1: Strain List

AM1003	*hml*Δ*::ADE1/hml*Δ*::ADE3 MAT**a**-LEU2-tel/MATa-inc hmr*Δ*::HPH FS2*Δ*::NAT/FS2 leu2/leu2-3,112 thr4 ura3-52 ade3::GAL::HO ade1 met13*	(5)
AM1229	AM1003, but *lys2*Δ	(6)
AM1284	AM1229, but *LYS2* at the 36kb position between *SED4* and *ATG15*	(6)

AM2048	AM1284, but *ura3-52*::pCORE	This study
AM2074	AM2048, but *ura3-52Δ*	This study
AM2889	AM2074, but *HPH*::*KANMX*	This study
AM2972	AM2889, but *MATα-inc::ura3-29* Ori1	This study
AM2973	AM2889, but *MATα-inc*::*ura3-29* Ori2	This study
AM2161	AM2889, but *thr4::ura3-29* Ori1	This study
AM2927	AM2889, but *thr4::ura3-29* Ori2	This study
AM2971	AM2889, but *HPH::ura3-29* Ori1	This study
AM2949	AM2889, but *HPH::ura3-29* Ori2	This study
AM4310	AM2972, but *rev3::BSD*	This study
AM3011	AM2973, but *rev3::BSD*	This study
AM2461	AM2161, but *rev3::BSD*	This study
AM2941	AM2927, but *rev3::BSD*	This study
AM3018	AM2971, but *rev3::BSD*	This study
AM3021	AM2949, but *rev3::BSD*	This study
AM3614	AM2973, but *KanMX::Bleo*^{r}	This study
AM3626	AM3614, but transformed with pSR419 empty vector (39)	This study
AM3629	AM3614, but transformed with pSR355 APOBEC3A (39)	This study
AM3529	AM2927, but *KanMX::Bleo*^{r}	This study
AM3584	AM3529, but transformed with pSR419	This study
AM3583	AM3529, but transformed with pSR355	This study
AM3617	AM2949, but *KanMX::Bleo*^{r}	This study

AM3632	AM3617, but transformed with pSR419	This study
AM3635	AM3617, but transformed with pSR355	This study
AM3647	AM3617, but *ung1Δ*	This study
AM3660	AM3647, but transformed with pSR419	This study
AM3663	AM3647, but transformed with pSR355	This study
AM3728	AM3647, but *rev3::BSD*	This study
AM4054	AM3728, but transformed with pSR419	This study
AM4055	AM3728, but transformed with pSR355	This study
AM4074	AM3617, but *mph1::KanMX*	This study
AM4109	AM4074, but empty vector	This study
AM4110	AM4074, but transformed with pSR355	This study
AM4076	AM3617, but *ubc13::kanMX*	This study
AM4112	AM4076, but transformed with pSR419	This study
AM4113	AM4076, but transformed with pSR355	This study
AM4212	AM3617, but *ntg2::kanMX*	This study
AM4239	AM4212, but transformed with pSR419	This study
AM4240	AM4212, but transformed with pSR355	This study
AM4281	AM4212, but *ntg1::BSD*	This study
AM4283	AM4281, but transformed with pSR419	This study
AM4284	AM4281, but transformed with pSR355	This study
AM4257	AM3617, but *apn1::kanMX*	This study
AM4267	AM4257, but transformed with pSR419	This study
AM4268	AM4257, but transformed with pSR355	This study

AM4215	AM3617, but *apn2::BSD*	This study
AM4241	AM4215, but transformed with pSR419	This study
AM4242	AM4215, but transformed with pSR419	This study
AM4260	AM4215, *apn1::kanMX*	This study
AM4269	AM4260, but transformed with pSR419	This study
AM4270	AM4260, but transformed with pSR355	This study
AM4321	AM3617, but *rad59::kanMX*	This study
AM4324	AM4321, but transformed with pSR419	This study
AM4325	AM4321, but transformed with pSR355	This study
AM4420	AM3617, but *mus81::kanMX*	This study
AM5281	AM4420, but transformed with pSR419	This study
AM5282	AM4420, but transformed with pSR355	This study
AM4417	AM3617, but *csm1Δ* using CRISPR-Cas9	This study
AM5283	AM4417, but transformed with pSR419	This study
AM5284	AM4417, but transformed with pSR355	This study
AM4418	AM3617, but *psy3Δ* using CRISPR-Cas9	This study
AM5285	AM4418, but transformed with pSR419	This study
AM5286	AM4418, but transformed with pSR355	This study
AM4490	AM4260, but *ntg1Δ, ntg2Δ* using CRISPR-Cas9	This study
AM5287	AM4490, but transformed with pSR419	This study
AM5288	AM4490, but transformed with pSR355	This study
AM5415	AM3617, but *pol3-01* using CRISPR-Cas9	This study
AM5434	AM5415, but transformed with pSR419	This study

AM5436	AM5415, but transformed with pSR355	This study
AM3025	AM2161, but *rad30::Bleo*^{r}	This study
AM3026	AM2927, but *rad30::Bleo*^{r}	This study
AM5397	AM4420, but *rad1::BSD*	This study
AM5408	AM4397, but transformed with pSR419	This study
AM5410	AM4397, but transformed with pSR355	This study

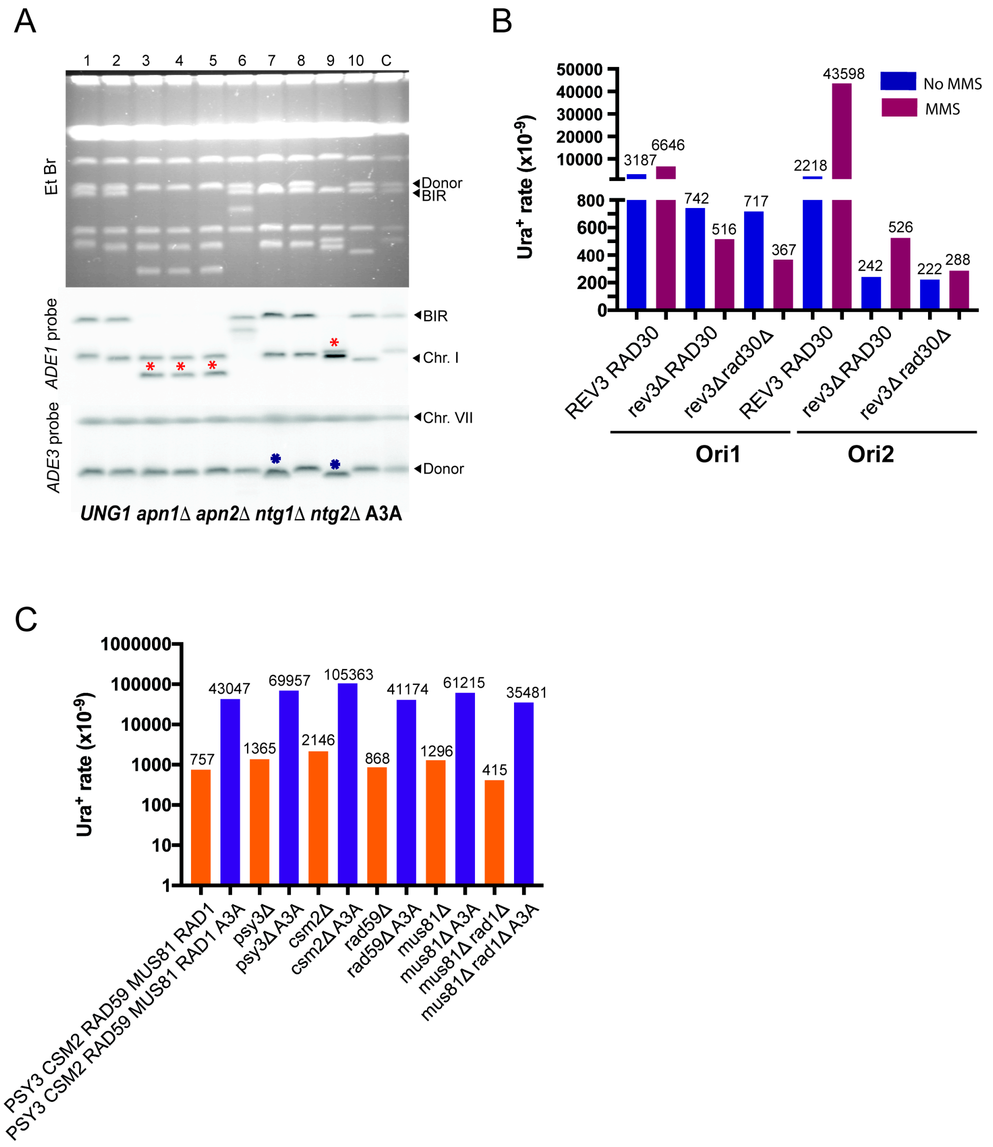

Supplementary Figure S1. **The role of base excision repair (BER), *REV3, RAD30,* and recombination genes in BIR/A3A-induced GCRs and mutagenesis. (A)** The effect of deleting BER genes on GCR. CHEF gel analysis of Ura^{+} BIR outcomes from *UNG1 ntg1Δ ntg2Δ apn1Δ apn2Δ* strains. Top: ethidium bromide-stained CHEF gels; middle: Southern blot hybridization with *ADE1*-specific probe and with *ADE3*-specific probe (bottom). Lanes labeled "C": BIR repair control. Red asterisks denote rearranged recipient (*ADE1*-containing) chromosome. Blue asterisks: rearranged donor (*ADE3*-containing) chromosome. **(B)** The role of *REV3* (encoding catalytic subunit of Pol ζ) and *RAD30* (encoding Pol η) on BIR/A3A-induced mutagenesis at Ori2 *ura3-29* at16kb position. **(C)** The effect of deleting recombination genes on BIR/A3A–induced mutagenesis. The rate of Ura^{+} mutations measured following BIR in the presence of A3A- or empty plasmid in strains bearing Ori2 *ura3-29* at 90kb position and deletions of various recombination-related genes. Absence of asterisk indicates no statistical difference between groups. See Supplementary Table S3 for p-values, description of statistical analysis, 95% CI of the medians, as well as for BIR efficiencies.

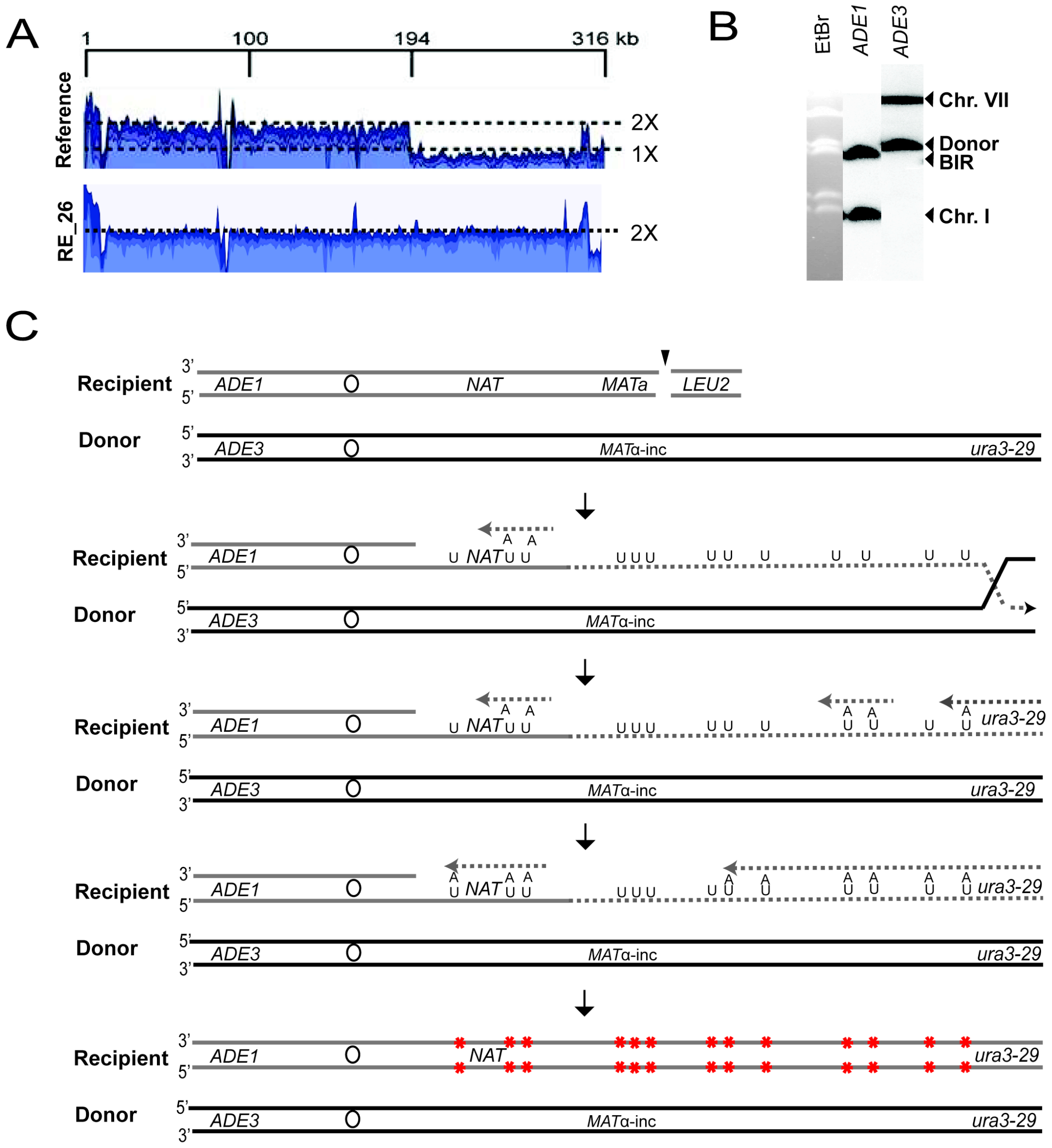

Supplementary Figure S2. Mutation cluster formation in RE_26 (*ung1Δ*). (A) Coverage of Illumina sequencing reads for RE_26 is increased two times for the chromosomal region located centromere distal to *MAT* (positions >190,180 bp) as compared to parental strain (reference). **(B)** Ethidium bromide stained CHEF gel electrophoresis (left). Middle and right: Southern blot analysis using *ADE1*-and *ADE3*-specific probes, respectively. **(C)** Proposed pathway leading to the formation of clustered mutations in RE_26. Broken *MAT***a**-chromosome (recipient) invades into the homologous chromosome (donor) and uracils (U) accumulate following A3A-mediated deamination of cytidines included into ssDNA accumulated behind the BIR bubble. During lagging strand synthesis, Pol δ inserts adenines across from uracils, which leads to C to T transitions (red asterisks) located in the recipient chromosome.

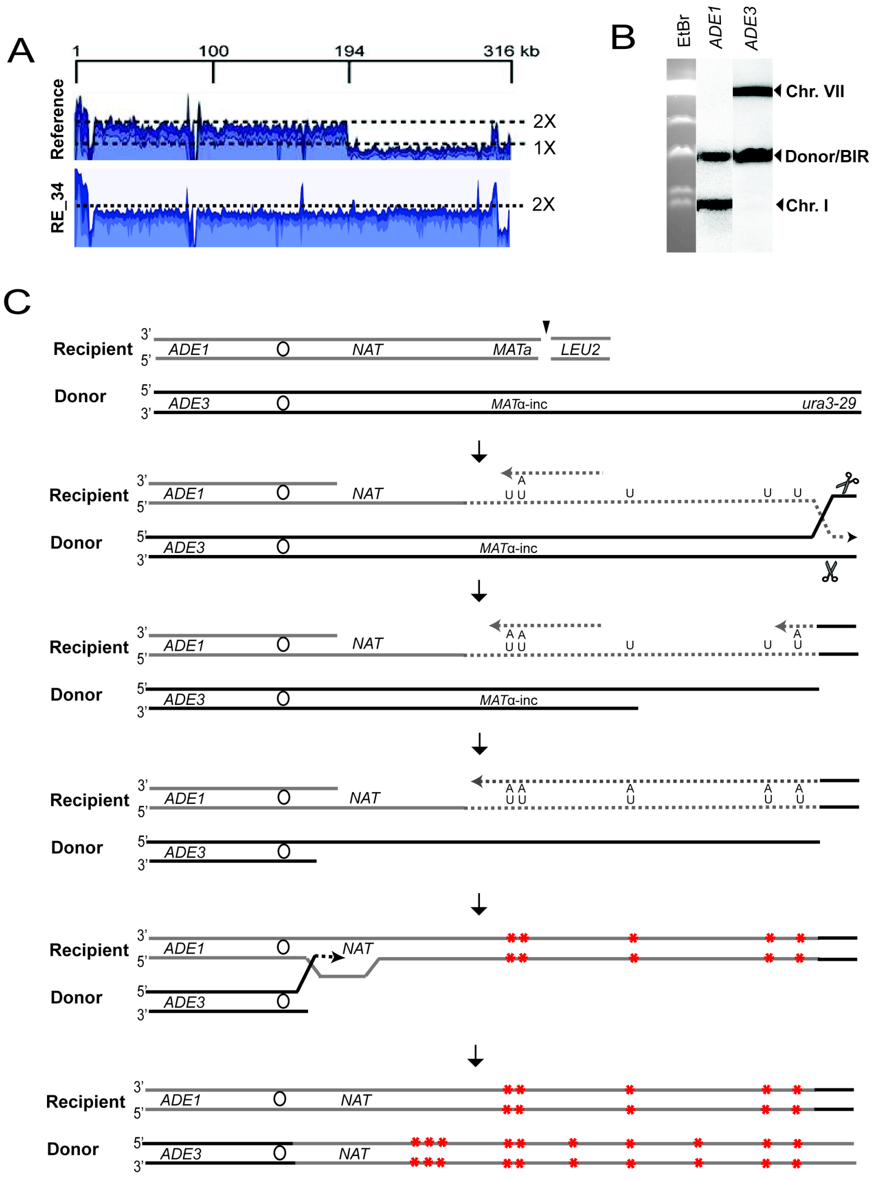

Supplementary Figure S3. Formation of RE_34 mutation cluster associated with GCR (*ung1Δ)*. (A) Coverage of Illumina sequencing reads for RE_34 as compared to parental strain (reference). **(B)** Ethidium bromide stained CHEF gel electrophoresis (left). Middle and right: Southern blot analysis using *ADE1*-and *ADE3*-specific probes, respectively. **(C)** Proposed pathway leading to the formation of clustered mutations and GCR in RE_34. Broken *MAT***a**-chromosome (recipient) invades into the homologous chromosome (donor) and uracils accumulate following cytidine deamination (U). BIR synthesis is interrupted near the end of the chromosome, which leads to the formation of a half-crossover (HC). The broken donor invades into the HC at the position located centromere proximal to *NAT* and initiates a secondary BIR synthesis that proceeds till the end of the chromosome giving rise to the repair outcome containing repaired recipient chromosome that is equal in size to the donor (B) and containing cluster including homozygous and heterozygous mutations (red asterisks).

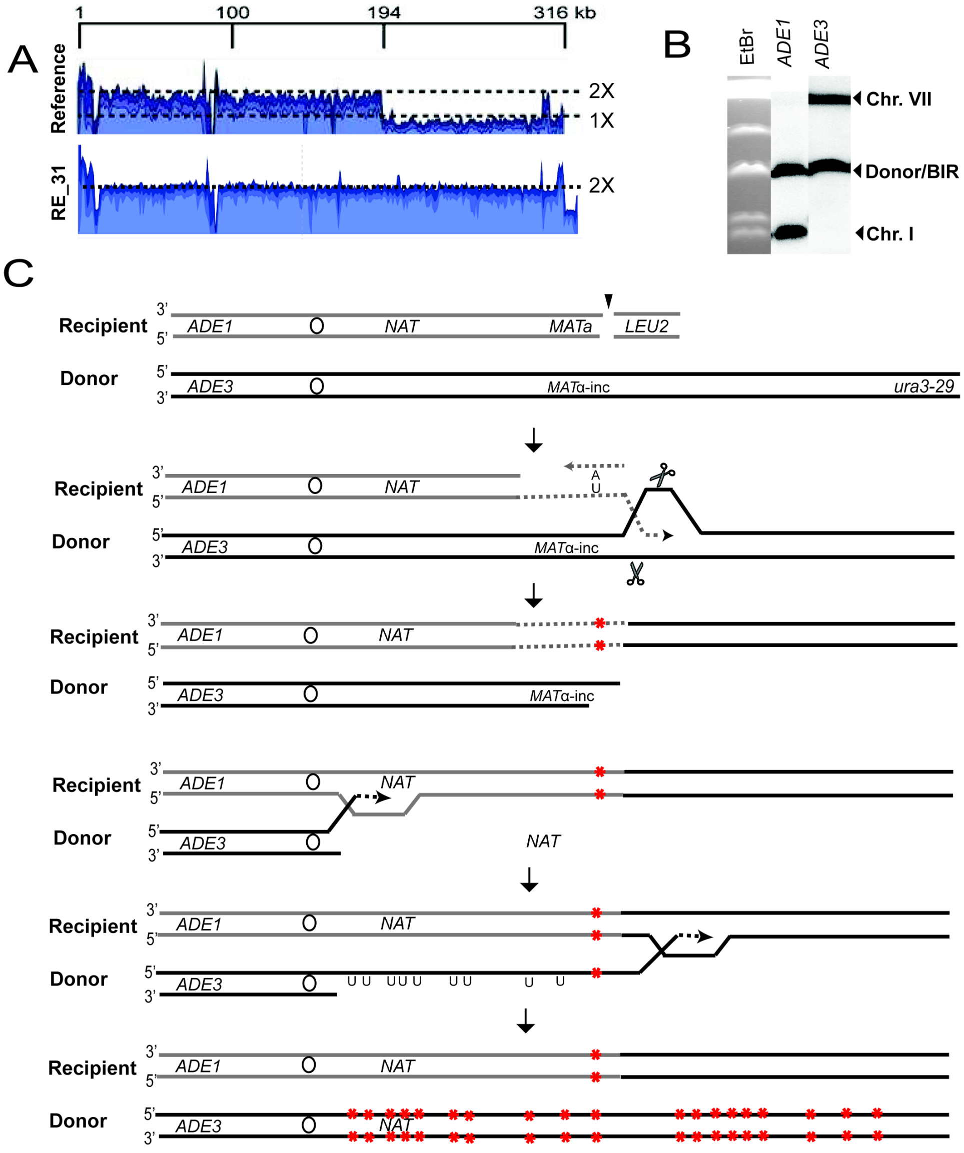

Supplementary Figure S4. Formation of RE_31 mutation cluster accompanied by GCR (*ung1Δ*). (A) Coverage of Illumina sequencing reads for RE_31 as compared to parental strain (reference). **(B)** Ethidium bromide stained CHEF gel electrophoresis (left). Middle and right: Southern blot analysis using *ADE1*-and *ADE3*-specific probes, respectively. **(C)** Proposed pathway leading to the formation of clustered mutations and GCRs in RE_31. The broken chromosome (recipient) invades into the homologous chromosome (donor). The progression of BIR bubble is interrupted early and resolution (scissors) occurs resulting in HC. The newly broken donor then invades into HC proximal to *NAT* and initiates a secondary BIR event leading to the accumulation of uracils (U). This results in the mutations (red asterisks) and also in rearranged donor that becomes similar in size to the recipient chromosome (see (B)).

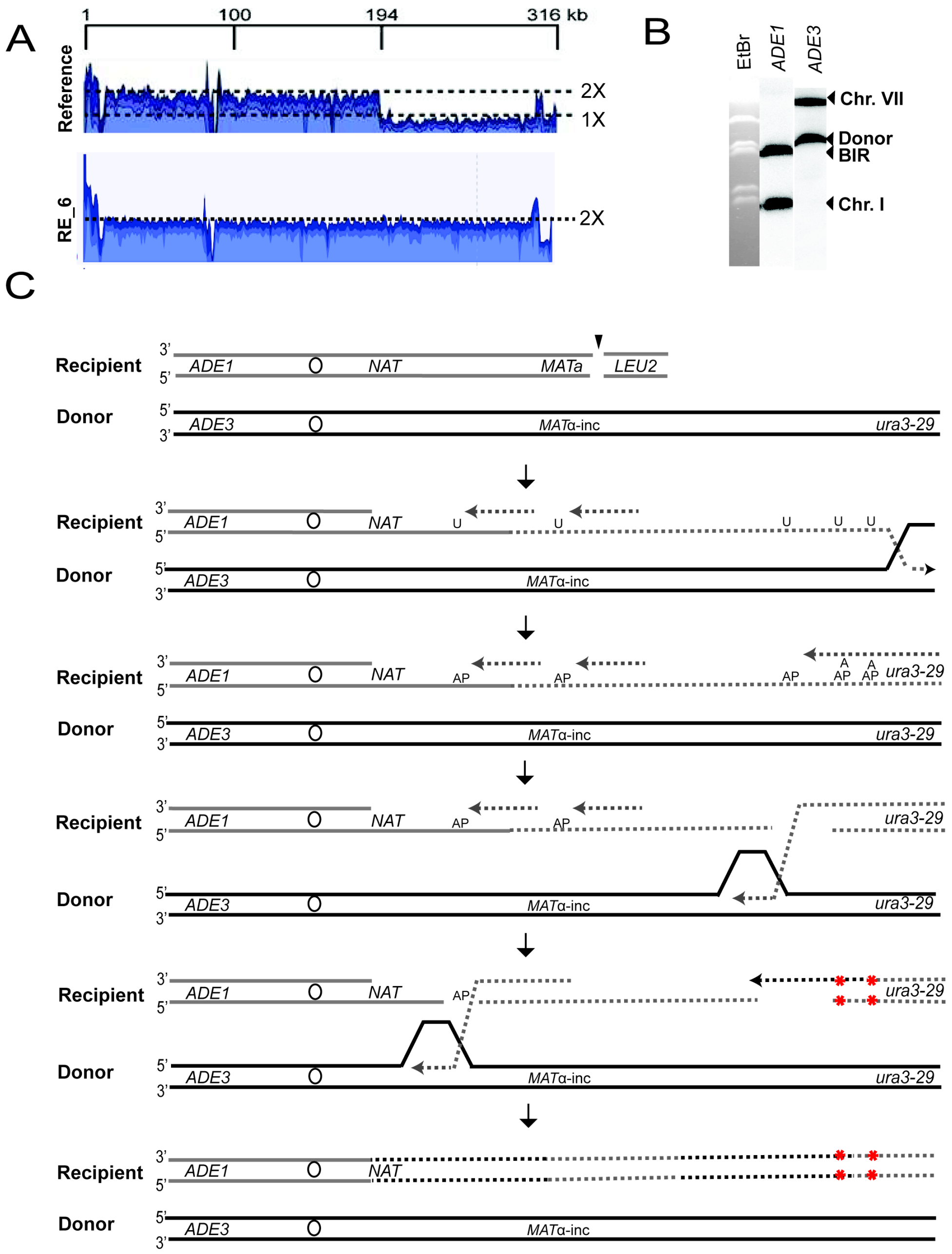

Supplementary Figure S5. Formation of RE_6 mutation cluster (*UNG1*). (A) Coverage of Illumina sequencing reads for RE_6 as compared to parental strain (reference). **(B)** Ethidium bromide stained CHEF gel electrophoresis (left). Middle and right: Southern blot analysis using *ADE1*-and *ADE3*-specific probes, respectively. **(C)** Proposed pathway of mutation cluster formation in RE_6. BIR is initiated following a DSB and strand invasion into homologous chromosome, and A3A induced lesions, uracils (U), accumulate in the ssDNA formed behind the BIR bubble. Uracils are excised by uracil glycosylase forming AP sites. During lagging strand synthesis, Pol δ stalls at AP sites leading to breakage and invasion of 3' ssDNA into homologous chromosome and initiation of DNA synthesis allowing to bypass AP sites accumulated in the leading strand of BIR. The newly synthesized bypass strand then dissociates and anneals to the leading strand of the recipient chromosome.

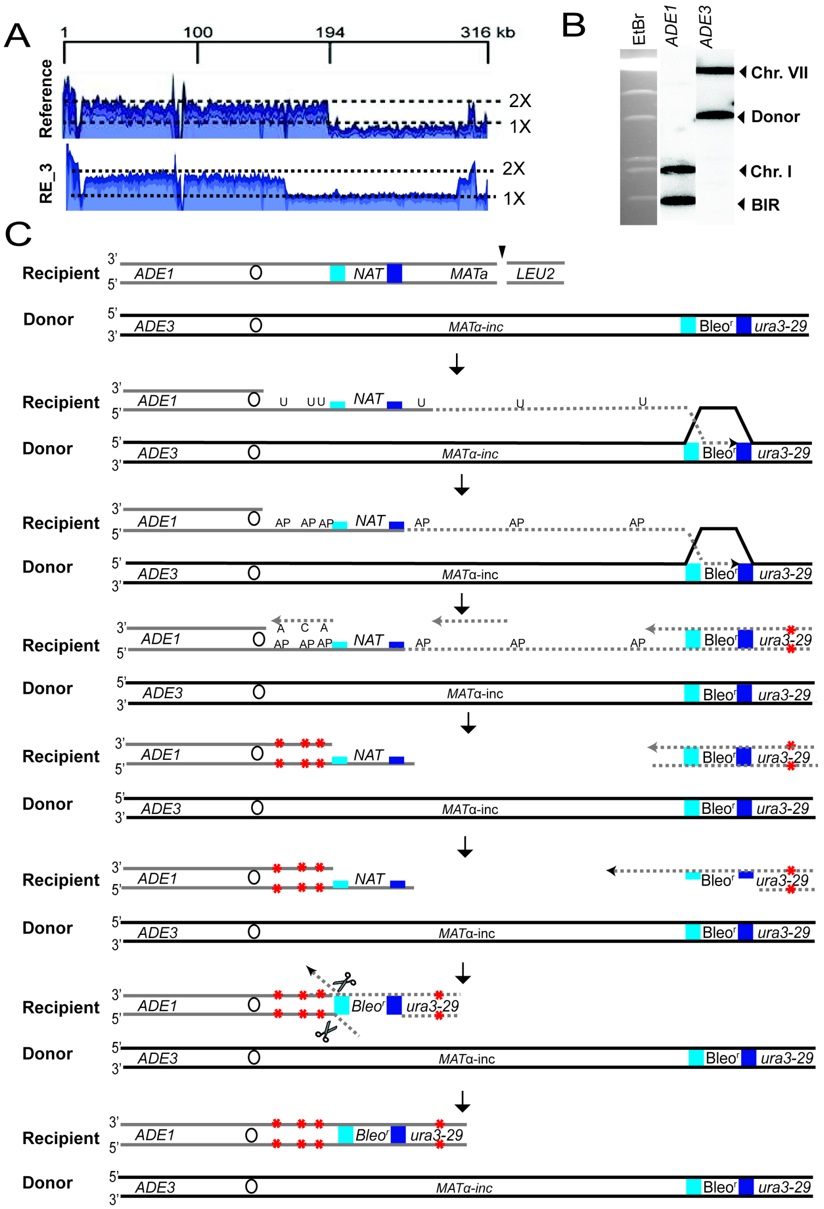

Supplementary Figure S6. Formation of RE_3 mutation cluster associated with GCR (*UNG1*). (A) Coverage of Illumina sequencing reads for RE_3 indicating formation of repaired recipient with a deletion between *NAT* (position 167kb of Chr. III) and *ura3-29* at 90kb (position 291kb of Chr. III). **(B)** Ethidium bromide stained CHEF gel electrophoresis (left). Middle and right: Southern blot analysis using *ADE1*-and *ADE3*-specific probes, respectively. **(C)** Proposed pathway explaining formation of clustered mutations in RE_3. BIR initiation occurs by invasion of broken chromosome into the homolog and copying that continues until the end of the chromosome. The newly synthesized ssDNA accumulates uracils (U) converted into abasic site (AP) by uracil glycosylase. Breakage at AP site leads to single-strand annealing (SSA) between the *TEF* sequences (~200bp direct repeats, depicted as blue rectangles) located at *NAT* and *BLEO*^{r} resulting in the repair outcome containing deletion. The non-homologous flaps following annealing are removed by endonucleases (scissors). Red asterisks denote mutations in a cluster.

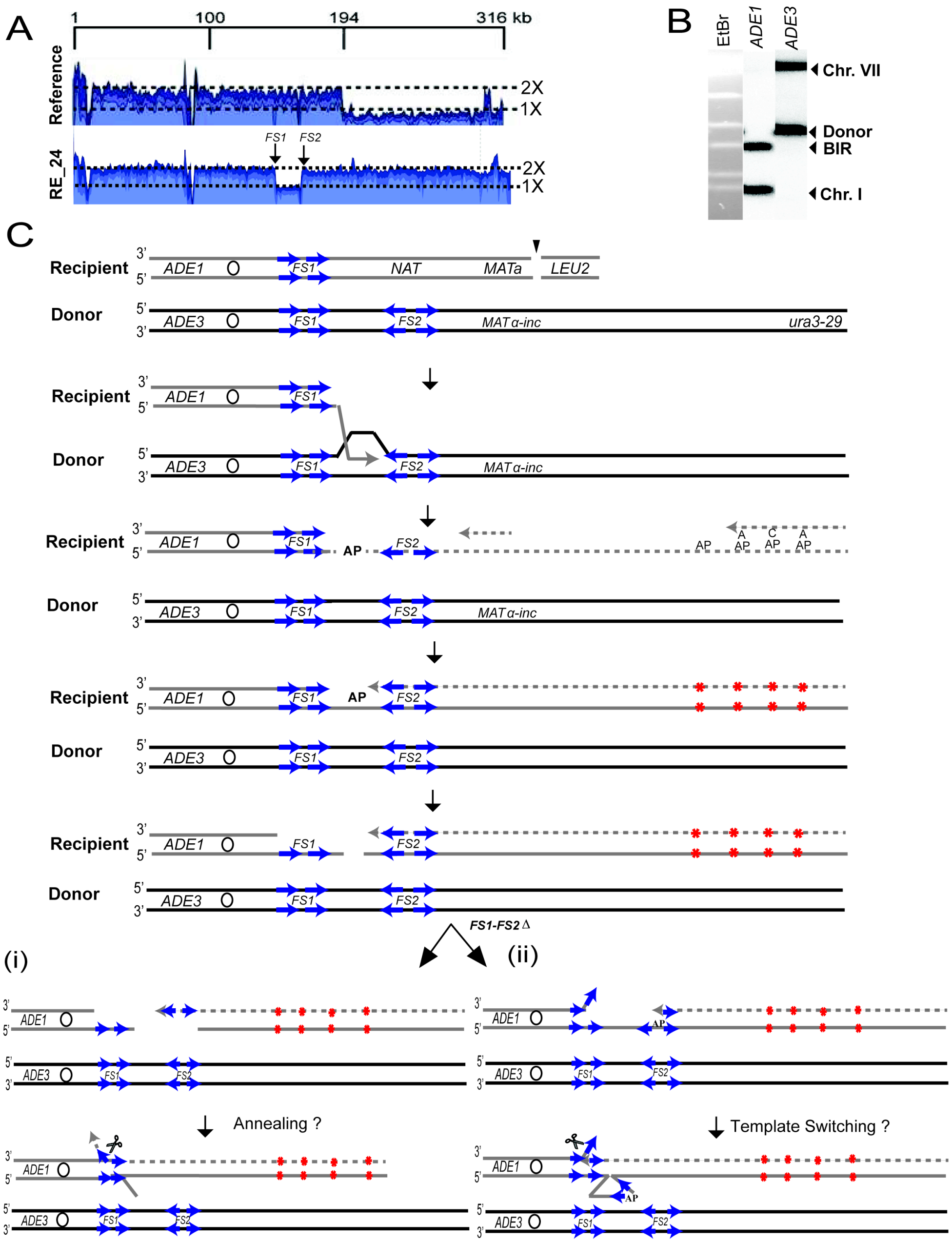

Supplementary Figure S7. Formation of RE_24 mutation cluster associated with GCR (*UNG1*). **(A)** Coverage of Illumina sequencing reads for RE_24. **(B)** Ethidium bromide stained CHEF gel electrophoresis (left). Middle and right: Southern blot analysis using *ADE1*-and *ADE3*-specific probes, respectively. **(C)** Proposed pathway leading to the formation of clustered mutations in RE_24. BIR is initiated by the invasion into the homologous chromosome, and leads to accumulation of lesions in ssDNA, giving rise to mutation clusters. **(i)** During lagging strand synthesis, DSBs are formed at AP site(s). SSA between Ty1 repeats in *FS1* and *FS2* leading to deletion of the region between *FS1* and *FS2*. This results in an outcome where the donor remains unchanged but the recipient is shorter in length (*FS1-FS2Δ*). **(ii)** Formation of deletion between *FS1* and *FS2* by template switching during lagging strand BIR synthesis initiated by the stalling of Pol δ at AP site

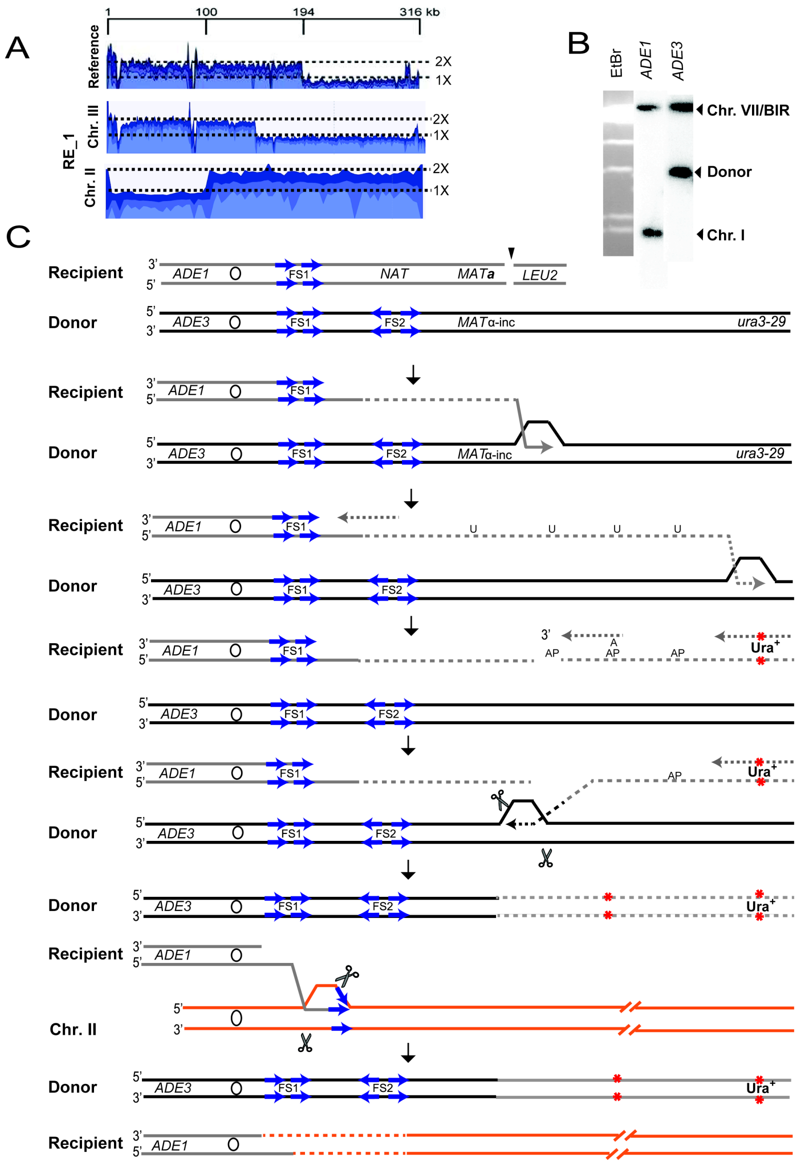

Supplementary Figure S8. Formation of RE_1 mutation cluster associated with GCR (*UNG1*). **(A)** Coverage of Illumina sequencing reads for RE_1 as compared to parental strain (reference) showing a 1X coverage along the BIR track on Chr. III and a duplication (2X) of a segment of chromosome II. **(B)** Ethidium bromide stained CHEF gel electrophoresis (left). Middle and right: Southern blot analysis using *ADE1*-and *ADE3*-specific probes, respectively. **(C)** Proposed pathway explaining formation of clustered mutations in RE_1. BIR is initiated following a DSB. The 3' end of the resected DNA end invades into the homologous template and copies until the end of the donor chromosome. The ssDNA formed behind the bubble accumulates A3A-produced uracils that are later transformed into abasic (AP) sites by uracil glycosylase. During lagging strand synthesis, the newly synthesized strand stalls at AP sites and following breakage at AP sites it re-invades into the donor, initiates DNA synthesis, which is interrupted resulting in the formation of HC. This results in the breakage of the donor chromosome, which then invades into an unmapped Ty or delta element in Chr. II. This invasion and brief synthesis is interrupted, which leads to the formation of a HC leading to translocation (and to the duplication of a fragment of Chr. II (see in (B)).

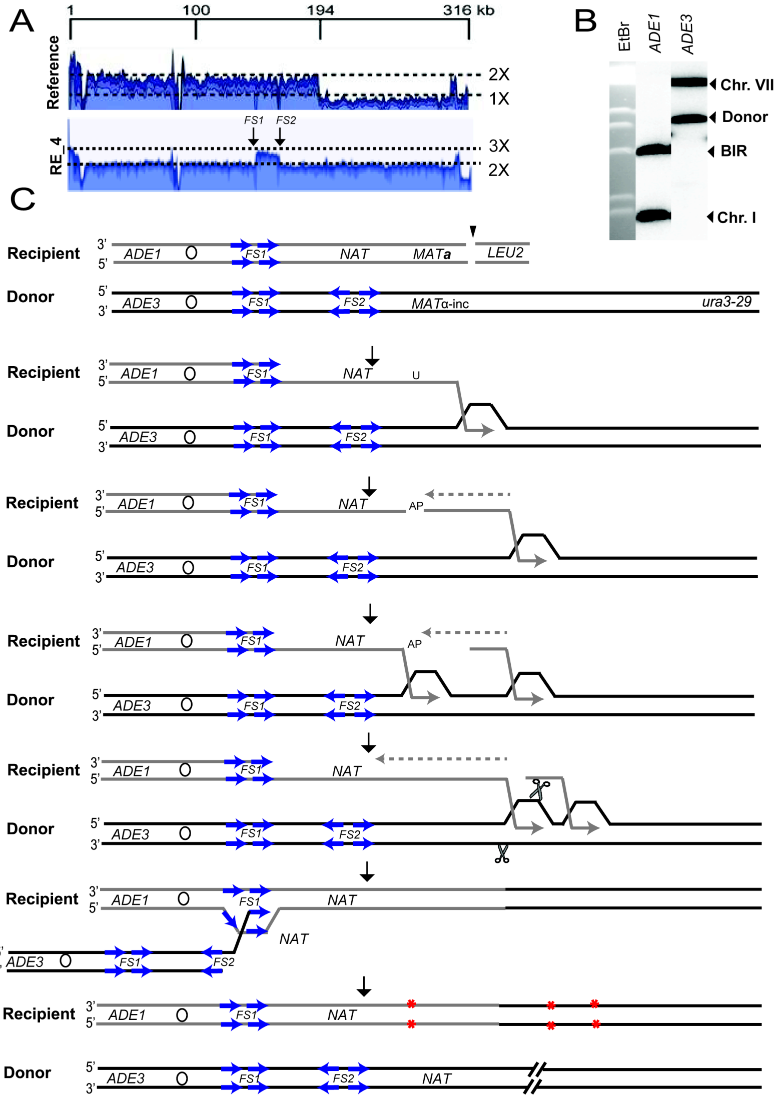

Supplementary Figure S9. Formation of RE_4 mutation cluster associated with GCR (*UNG1*). (A) Coverage of Illumina sequencing reads for RE_4 as compared to parental strain (reference) showing 3X coverage of the region in between *FS1* and *FS2* elements **(B)** Ethidium bromide stained CHEF gel electrophoresis (left). Middle and right: Southern blot analysis using *ADE1*-and *ADE3*-specific probes, respectively. **(C)** Proposed pathway explaining formation of clustered mutations in RE_4. DSB is introduced in the recipient chromosome and following resection, BIR is initiated by invasion into the donor chromosome. This synthesis extends until the end of the donor chromosome and accumulates DNA lesions along the ssDNA. The excision of uracils formed by A3A by uracil glycosylase results in the formation of abasic (AP) sites. The lagging strand synthesis stalls at an AP site and following breakage the centromere proximal end of the DSB re-invades into the template initiating a "secondary" BIR event. Collision of the "primary" and "secondary" BIR bubbles results in BIR stalling and the subsequent formation of a half-crossover. *FS2* of the newly broken donor then invades into the *FS1* repeat of the recipient and re-initiates DNA synthesis until the end of the chromosome resulting in the formation of a donor chromosome that is larger in size than the initial donor length (refer to (B)).

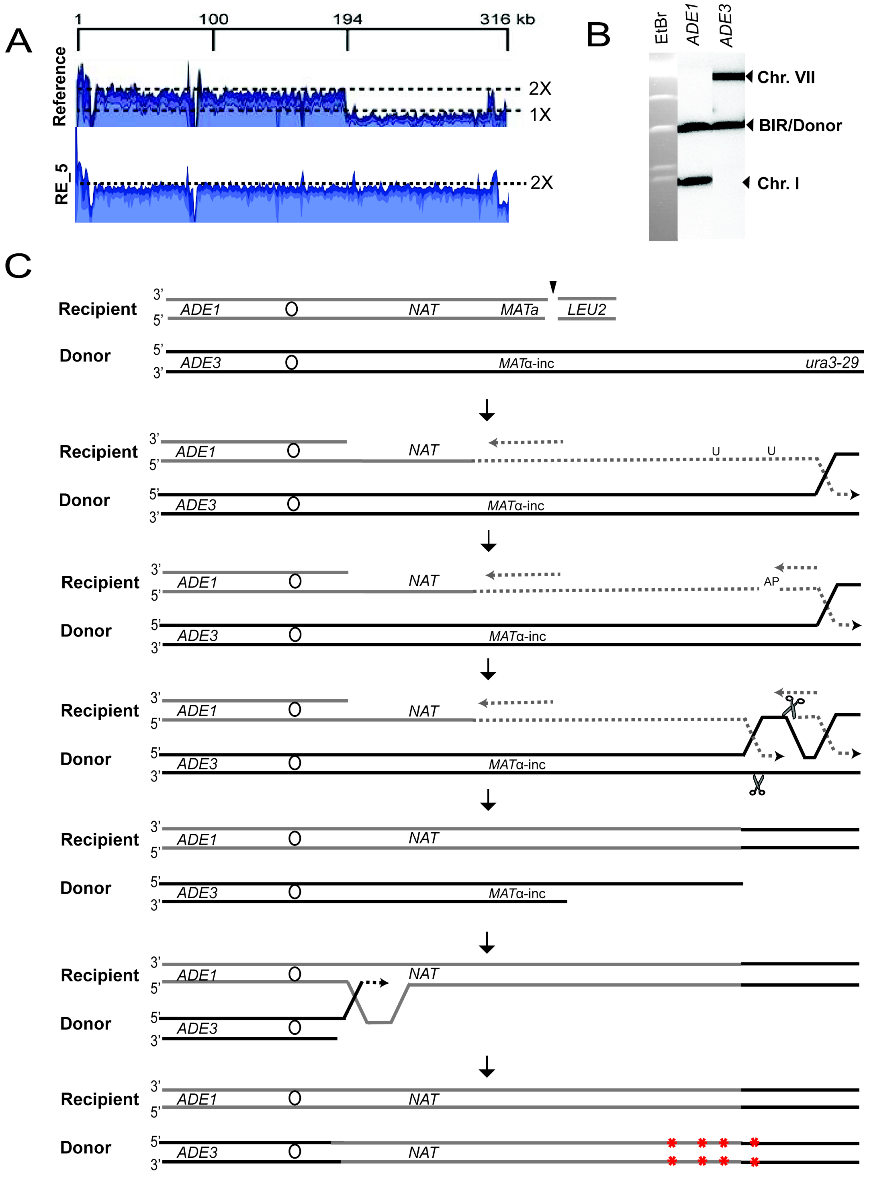

Supplementary Figure S10. Formation of RE_5 mutation cluster associated with GCR (*UNG1*). (A) Coverage of Illumina sequencing reads for RE_5 as compared to parental strain (reference). **(B)** Ethidium bromide stained CHEF gel electrophoresis (left). Middle and right: Southern blot analysis using *ADE1*-and *ADE3*-specific probes, respectively. **(C)** Proposed pathway explaining formation of clustered mutations in RE_5. A DSB is introduced in the recipient and the broken chromosome initiates BIR by invading into the donor chromosome. During BIR synthesis, long ssDNA accumulates behind the bubble, and AP sites accumulate following deamination of cytidines and conversion of the resulting uracils into AP sites by uracil glycosylase. During lagging strand synthesis, a broken DNA end formed at an AP site re-invades into the donor behind the bubble initiating “secondary” BIR. Collision of the “primary” and “secondary” BIR bubbles results in the formation of a HC breaking the donor chromosome. This newly broken chromosome re-invades the recipient and new mutations are formed during this second round of BIR synthesis resulting in the formation of heterozygous mutation cluster
